# The male germ unit association is independently regulated of GUM in *Arabidopsis thaliana*

**DOI:** 10.1002/pld3.624

**Published:** 2024-07-29

**Authors:** Abdur Rauf, Anbang Wang, Yujia Li, Zhihao Lian, Shouxing Wei, Kashmala Jabbar, Muhammad Wisal, Ikramullah Khan, Muhammad Khalid, Jingyang Li

**Affiliations:** ^1^ National Key Laboratory for Tropical Crop Breeding, Tropical Crop Genetic Resources Institute Chinese Academy of Tropical Agricultural Sciences Sanya/Haikou Hainan China; ^2^ Garden Campus, Department of Botany Abdul Wali Khan University Mardan KP Pakistan; ^3^ Department of Genetic and Genome Biology University of Leicester UK; ^4^ Hainan Banana Healthy Seedling Propagation Engineering Research Center, Haikou Experimental Station Chinese Academy of Tropical Agricultural Sciences Haikou Hainan China; ^5^ GPGGCM Abdul Wali Khan University Mardan KP Pakistan; ^6^ Department of Biology, College of Science, Mathematics and Technology Wenzhou‐Kean University Wenzhou China

**Keywords:** *A. thaliana*, generative cell, germ unit malformed, male germ unit, sperm cell

## Abstract

Cytoplasmic projections (CPs) formed by the generative and sperm cells link the male gametes with the vegetative cell (VC) nucleus, which are required to build the male germ unit (MGU) assemblage in the angiosperm pollen grain. As molecular and genetic controls underlying CP development and formation of the MGU are unknown, it was hypothesized that physical association between germ cells and the VC nucleus might be lost in *germ unit malformed* (*gum*) mutants or in those which either block generative cell (GC) division or that additionally prevent gamete differentiation. In vivo, analysis of marked cellular components demonstrated a linkage of sperm cells (SCs) and the VC nucleus in *gum* mutant alleles despite their increased physical separation. Similarly, for several independent classes of bicellular pollen mutants, undivided GCs were associated with the VC nucleus like GCs in wild‐type pollen. We conclude that the early formation of GC CPs to establish the MGU is regulated independently of DUO1‐DAZ1 and DUO3 transcription factors as well as cyclin‐dependent kinase function (CDKA;1). As the absence of cytoplasmic protrusion was expected in the *gum* mutants in Arabidopsis, early histological studies reported temporal disappearance of cytoplasmic protrusion in several organisms. Our findings demonstrated the striking importance of live imaging to verify the broad conservation of the persistent MGU contact in all the angiosperms and its important role in successful double fertilization.

## 1 INTRODUCTION

The male germline cells (generative cell [GC]/sperm cell [SC]) in developing pollen of angiosperms form fine cytoplasmic projections (CPs) which physically link the male gametes with the pollen vegetative cell (VC) nucleus to create a structural assemblage, known as the male germ unit (MGU) (Dumas et al., 1985). Evidence for the MGU was first observed in pollen of cotton (Jensen & Fisher, 1968), and one of the SCs of the pair is linked with the VC nucleus by a CP (Braun & Winkelmann, 2016; Goto et al., 2020; Kliwer & Dresselhaus, 2010; McConchie, Hough, & Knox, 1987; McCue et al., 2011; Zee & Ye, 2000; Zhou & Meier, 2014). The physical association between the GC and VC nucleus has been documented in early to mid‐bicellular stage pollen (Table 1). This association is observed in the majority of bicellular pollen species, which shed bicellular pollen at anthesis and within the pollen tube (Table 1). Similarly, the MGU association has also been observed in many tricellular pollen species at different developmental stages (Table 2). The presence of the MGUs in angiosperm pollen and their presumptive importance in double fertilization is now largely accepted (reviewed in Mogensen, 1992; Dumas et al., 1998; McCue et al., 2011).

**TABLE 1:** Review of evidence for male germ unit association among bicellular pollen species. The male germ unit has been documented in 49 out of 55 bicellular pollen species studied at the developmental stages indicated. The male germ unit was not detected only in two studies highlighted in gray (study IDs. 53 and 54) of *Helleborus foetidus* (Heslop‐Harrison et al., 1986) and *Haemanthus katherinae* (Sanger & Jackson, 1971). (BCP, bicellular pollen; TCP, tricellular pollen; GC generative cell; SC sperm cell; DIC, differential interference contrast; LM, light microscopy; EM, electron microscopy; TEM, transmission electron microscopy; CLSM, confocal laser scanning microscopy; IF, immunofluorescence; α, alpha; FM, fluorescence microscopy; LM, light microscopy; FCM, flow cytometry; DAPI, 4′,6‐diamidino‐2‐phenylindole; MT, microtubules; MF, microfilament; YFP, yellow fluorescent protein; TBO, toluidine blue; DiOC6, 3,3‐dihexyloxacarbocyanine iodide; FISH, fluorescence in situ hybridization).

ID no.	Species	Family	Stages analyzed	Early‐mid BCP	Late BCP	TCP	Pollen tube	Technique	Ref
1	*Nicotiana tabacum*	Solanaceae	Microspore to fertilization	Yes	Yes	Yes	Yes	Light and epifluorescence (CLSM)	Oh et al. (2010)
2	*N. tabacum*	Solanaceae	GC to SCs formation in pollen tube	NA	Yes	Yes	Yes	EM and serial TEM	Yu and Russell (1994)
3	*N. tabacum*	Solanaceae	Mature pollen inside pollen tube	NA	Yes	Yes	Yes	Immunofluorescence germline MT (antitubulin) and Hoechst staining	Palevitz (1993)
4	*N. tabacum*	Solanaceae	GC division inside pollen tube	NA	Yes	NA	Yes	IF and epifluorescence microscopy of aniline blue and DAPI/CLSM	Laitiainen et al. (2002)
5	*N. tabacum*	Solanaceae	Mature pollen inside pollen tube	NA	NA	NA	Yes	Epifluorescence DAPI	Tian et al. (2005)
**6**	*N. tabacum* and *Nicotiana alata*	Solanaceae	GC development and division inside pollen tube	NA	Yes	Yes	Yes	Immunofluorescence germline MT (antitubulin)/MFs, IF and epifluorescence microscopy DAPI	Åström et al. (1995)
7	*N. tabacum*	Solanaceae	SCs formation in pollen tube	NA	NA	Yes	Yes	TEM, 3‐D serial reconstruction and quantitative cytology	Yu et al. (1992)
8	*N. tabacum*	Solanaceae	Germinating pollen tube	NA	NA	NA	Yes	Immunofluorescence germline MT/MFs, IF and epifluorescence microscopy DAPI	Åström et al. (1991)
9	*N. tabacum*	Solanaceae	SCs released from pollen tube	NA	NA	Yes	NA	Light and scanning electron microscopy	Tian et al. (1998)
10	*N. tabacum*	Solanaceae	Isolated sperm cells from germinating pollen tube	NA	NA	Yes	Yes	Light microscopy	Tian and Russell (1998)
11	*N. tabacum*	Solanaceae	Early to late bicellular	No	Yes	Yes	NA	Photomicroscopy of live SC	Tian et al. (2001)
12	*Hippeastrum vittatum*	Liliaceae	Mature pollen	NA	Yes	NA	NA	EM and 3‐D reconstruction	Mogensen (1986)
13	*Populus deltoides*	Salicaceae	GC at anthesis to SCs formation in pollen tube	NA	Yes	Yes	Yes	DAPI epifluorescence, light and EM	Rougier et al. (1991)
14	*Hyacinthus orientalis*	Asparagaceae	Late BCP to SCs formation in pollen tube	NA	Yes	Yes	Yes	Immunofluorescence germline MT/F‐actin using CLSM and EM	Del Casino et al. (1992)
15	*Convallaria majalis*	Asparagaceae	GC division and SC formation inside pollen tube	NA	Yes	Yes	Yes	Immunofluorescence of α‐tubulin, DAPI and CLMS	Del Casino et al. (1999)
16	*Tropaeolum majus*	Tropaeolaceae	Early to mature pollen at dehiscence and inside pollen tube	Yes	Yes	Yes	Yes	Epifluorescence DAPI, TBO, and EM	Niu et al. (1999)
17	*Petunia hybrida*	Solanaceae	Mature pollen at dehiscence and inside pollen tube	NA	Yes	Yes	Yes	3‐D reconstruction quantitative TEM	Wagner and Mogensen (1988)
18	*Medicago sativa*	Fabaceae	Early and mature pollen grain	Yes	Yes	NA	NA	3‐D reconstruction and TEM	Shi et al. (1991)
19	*Gasteria verrucosa*	Asphodelaceae	Microspore to SCs formation inside pollen tube	Yes	Yes	NA	NA	Microspectrophotometry, Immunolocalization and EM	Van Lammeren et al. (1985)
20	*Pisum sativum*	Fabaceae	GC inside pollen tube	NA	NA	NA	Yes	Lmmunofluorescence microscopy of anti‐RoplPs	Lin et al. (1996)
21	*Solanum lycopersicum*	Solanaceae	GC and SC during pollen germination and tube growth	NA	Yes	Yes	Yes	DIC, DAPI	Lu et al. (2015)
22	*Lilium longiflorum* and *Tulipa gesneriana*	Liliaceae	Uninucleate microspores to mature pollen	NA	Yes	NA	NA	Epifluorescence microscopy by antiserum and DAPI	Tanaka et al. (1998)
23	*L. longiflorum* and *Brassica napus*	Liliaceae/Brassicaceae	GC and SC during in vivo grown pollen tubes	NA	Yes	Yes	Yes	Immunogold labelling, light microscopy Hoechst 33258 and monoclonal antibodies (MAbs)	Blomstedt (1993)
24	*M. sativa*	Fabaceae	GC development in pollen tube	NA	Yes	NA	NA	3‐D reconstruction and TEM	Zhu et al. (1990)
25	*Gossypium hirsutum*	Malvaceae	Pollen grain before and after germination	Yes	Yes	NA	NA	Light and EM	Jensen et al. (1968)
26	*G. hirsutum*	Malvaceae	GC inside pollen tube	NA	NA	Yes	Yes	Light and EM	Jensen and Fisher (1970)
27	*Ornithogalum virens*	Asparagaceae	Mature pollen at anthesis and inside pollen tube	NA	Yes	Yes	Yes	Immunofluorescence germline MT using conventional and CLSM, DAPI	Banaś et al. (1996)
28	*Tradescantia virginiana*	Commelinaceae	Mature pollen and sperm cells in germinating pollen tube	NA	Yes	Yes	Yes	Antitubulin immunofluorescence of germline MT, rhodamine‐phalloidin and DAPI	Palevitz and Cresti (1988)
29	*Cyphomandra betacea*	Solanaceae	GC to SCs formation inside pollen tube	NA	Yes	Yes	Yes	Light and EM	Hu and Yu (1988)
30	*Leucojum aestivum*	Amaryllidaceae	Microsporogenesis, microgametogenesis and in vitro pollen tube growth	No	Yes	Yes	Yes	Light and fluorescence	Ekici and Dane (2012)
31	*Rhododendron anagalliflorum, R.Laetum* and *R.Lochae*	Ericaceae	Early to late bicellular (inside pollen tube)	NA	Yes	Yes	Yes	3‐D reconstruction, TEM and light microscopy	Theunis et al. (1985)
32	*Cymbidium goeringii*	Orchidaceae	Early to anthesis	Yes	Yes	NA	NA	TEM, 3‐D serial reconstruction and quantitative cytology	Yu and Russell (1992)
33	*Eucalyptus globulus*	Myrtaceae	Mature pollen	NA	Yes	NA	NA	Light (LM), scanning electron (SEM) and transmission electron microscopy (TEM)	Eliseu and Dinis (2008)
34	*Luehea divaricata*	Malvaceae	Early to anthesis	No	Yes	NA	NA	Transmission electron microscopy (TEM)	Lattar et al. (2012)
35	*Cyrtandra pendula*	Gesneriaceae	Early to late BCP pollen	Yes	Yes	NA	NA	TEM	Luegmayr (1993)
36	*Cyrtanthus mackenii*	Amaryllidaceae	GC division inside pollen tube	NA	Yes	Yes	Yes	Flow cytometry (FCM), DAPI and immunolocalization	Hirano and Hoshino (2010)
37	*Alstroemeria aurea*	Alstroemeriaceae	In vitro growth of mature pollen grain (GC and SC inside pollen tube)	NA	Yes	Yes	Yes	DAPI and FCM (flow cytometry)	Hirano and Hoshino (2009)
38	*Aechmea fasciata*	Bromeliaceae	Mature pollen at anthesis and MGU movement in pollen tube	NA	Yes	Yes	Yes	Light and fluorescence (DAPI) microscopy	Vervaeke et al. (2005)
39	*Abelia spathulata, Campsis grandiflora, Erythrina variegata, Limonium sinuatum*, and *Tecoma capensis*	Caprifoliaceae/	Mature pollen at anthesis	NA	Yes	Yes	NA	Fluorescence microscopy DAPI, TBO, and DiOC_6_	Saito et al. (2002)
		Bignoniaceae/							
		Fabaceae/Plumbaginaceae/Bignoniaceae							
40	*H. orientalis*	Asparagaceae	Mature pollen germination and GC division into SC inside pollen tube	NA	Yes	Yes	Yes	Immunolocalisation of 5‐Bromouracil, FISH, DAPI, CLSM	Zienkiewicz et al. (2011)
41	*H. orientalis*	Asparagaceae	Mature pollen at anthesis and GC division into SC inside pollen tube	NA	Yes	Yes	Yes	Immunolocalisation of 5‐methylcytosine (5mC), acetylated histone 4 (ACH4) and histone deacetylase 1 (HDT1)/DAPI/CLSM	Kozłowska et al. (2016)
42	*Torenia fournieri*	Linderniaceae	Isolated SC from germinating pollen tube	NA	NA	Yes	Yes	DIC and light (fluorescein diacetate‐FDA) microscopy	Chen et al. (2006)
43	*Colobanthus quitensis*	Caryophyllaceae	Mature pollen	NA	NA	Yes	NA	Light (LM) and transmission electron microscopy (TEM)	Giełwanowska et al. (2011)
44	*Mimulus aurantiacus*	Phrymaceae	Pollen tube growth	NA	NA	NA	Yes	EM	Ekici et al. (2009)
45	*Vitis vinifera*	Vitaceae	Mature pollen	NA	Yes	NA	NA	Scanning electron (SEM) and transmission electron microscopy (TEM)	Cresti and Ciampolini (1999)
46	*Polystachia pubescens*	Orchidaceae	Early to late BCP pollen	Yes	NA	NA	NA	Light (LM) and transmission electron microscopy (TEM)	Schlag and Hesse (1992)
47	*Papaver rhoeas*	Papaveraceae	Germinating pollen tube	NA	NA	NA	Yes	Light and CLSM using calcium green‐1	Straatman et al. (2001)
48	*Endymion non‐scriptus*	Asparagaceae	Mature pollen	NA	Yes	NA	NA	EM	Burgess (1970)
49	*T. virginiana, N. tabacum* and *Rhododendron laetum*	Commelinaceae/Solanaceae/Ericaceae	GC in pollen tube	NA	Yes	Yes	Yes	Immunolocalizations, Hoechst 33258 and N‐phenylenediamine nuclear staining	Palevitz and Liu (1992)
50	*Acacia retinodes*	Fabaceae	Early to GC formation in mature pollen	NA	Yes	NA	NA	Light microscopy and cytological analysis	McCoy and Knox (1988))
51	*Clivia nobilis*	Amaryllidaceae	In vitro culture of microspore to sperms formation in pollen tube	NA	Yes	Yes	Yes	Time‐lapse cine‐micrography and cytochemical observations on adenosine triphosphatase (ATPase)	Pei‐hua (1988)
	*Amaryllis vittata*								
52	*Crocus biflorus*	Iridaceae	Mature pollen	NA	Yes	NA	NA	DAPI epifluorescence, light and EM	Caiola et al. (1993))
53	*H. foetidus*	Ranunculaceae	GC at anthesis (hydrated/dehydrated) to pollen tube growth	No	No	No	No	Optical sectioning, DIC and DAPI epifluorescence	Heslop‐Harrison et al. (1986)
54	*H. katherinae*	Amaryllidaceae	Microspore to anthesis	No	No	No	NA	Light and EM	Sanger and Jackson (1971)
55	Petunia cultivars	Solanaceae	Mature pollen at dehiscence and inside pollen tube	NA	Yes	Yes	Yes	DAPI epifluorescence, light and EM	Hoshino et al. (2016)

**TABLE 2:** Review of evidence for male germ unit association among tricellular pollen species. The male germ unit has been documented in all 46 tricellular pollen species studied at the developmental stages indicated. The male germ unit was not detected in nine studies (study IDs. 6, 25, 27–28, 30, 33, 36–37, 40) that included those on *Arabidopsis thaliana* (Derksen et al., 2002; Kuang & Musgrave, 1996; Owen & Makaroff, 1995; Yamamoto et al., 2003)*, Zea mays* (Rusche & Mogensen, 1988), and *Hordeum vulgare* (Mogensen & Rusche, 1985), but independent studies have subsequently confirmed the presence of the male germ unit in all three species (Hamamura et al., 2011; Kliwer & Dresselhaus, 2010; Mogensen & Wagner, 1987; Reňák et al., 2012).

ID no.	Species	Family	Stages analyzed	Early‐mid BCP	Late BCP	TCP	Pollen tube	Technique	Ref
1	*Plumbago zeylanica*	Plumbaginaceae	GC to SC formation at five‐developmental stages	Yes	Yes	Yes	NA	3‐D reconstruction of TEM and quantitative analysis	Russell and Strout (2005)
2	*P. zeylanica*	Plumbaginaceae	Mature pollen grain and pollen tube germination	NA	NA	Yes	Yes	Light and EM	Russell and Cass (1981)
3	*P. zeylanica*	Plumbaginaceae	Mature pollen grain from newly open flower	NA	NA	Yes	NA	3‐D reconstruction, quantitative cytology, and TEM	Russell (1984)
4	*P. zeylanica*	Plumbaginaceae	Mature pollen grain	NA	NA	Yes	NA	Protein 2D‐PAGE	Geltz and Russell (1988)
5	*Z. mays*	Poaceae	GC to SC formation in pollen tube	Yes	Yes	Yes	Yes	Live cell imaging of germline MT (α‐tubulin‐YFP)	Kliwer and Dresselhaus (2010)
6	*Z. mays*	Poaceae	Mature pollen grain	NA	NA	No	NA	3‐D reconstruction, quantitative cytology and TEM	Rusche and Mogensen (1988)
7	*Z. mays*	Poaceae	Isolated SC from open flower	NA	NA	Yes	NA	Light (LM), scanning electron (SEM) and transmission electron microscopy (TEM)	Dupuis et al. (1987)
8	*Z. mays*	Poaceae	Mature pollen (sperm cells)	NA	NA	Yes	NA	DAPI, EM, and 3‐D reconstruction of serial thin sections	McConchie, Hough, and Knox (1987)
9	*Secale cereale*	Poaceae	Mature pollen grain	NA	NA	Yes	NA	EM and 3‐D reconstruction	Mogensen and Rusche (2000)
10	*Oryza sativa*	Poaceae	5‐stages (24‐phases) microspore to anthesis	Yes	Yes	Yes	NA	Immunofluorescence germline MT, CLMS	Zee and Ye (2000)
11	*Euphorbia dulcis*	Euphorbiaceae	Young to mature pollen grain (development of GC and SC)	NA	Yes	Yes	NA	TEM and fluorescence optical microscopy (DAPI)	Murgia et al. (1986)
12	*E. dulcis*	Euphorbiaceae	Mature pollen grain	NA	NA	Yes	NA	TEM	Murgia and Wilms (1987)
13	*Brassica napus*	Brassicaceae	Microspore to anthesis	No	No	Yes	NA	DAPI, light, and EM	Murgia et al. (1991))
14	*B. napus*	Brassicaceae	Mature pollen (sperm cell formation)	NA	NA	Yes	NA	EM	Charzynska et al. (1989)
15	*B. napus*	Brassicaceae	Germination of isolated microspore into bicellular and tricellular pollen	No	Yes	Yes	NA	Immunofluorescence of germline with TRITC (red), FITC (green), CLMS, DIC, DAPI, and DiOC_6_	Dubas et al. (2012)
16	*Spinacia oleracea*	Amaranthaceae	Isolated SC from open flower	NA	NA	Yes	NA	Phase contrast microscopy	Theunis and Van Went (1989)
17	*S. oleracea*	Amaranthaceae	Mature pollen grain	NA	NA	Yes	NA	3‐D reconstruction, CLSM	Theunis et al. (1991)
18	*S. oleracea*	Amaranthaceae	In vivo growth of SC in pollen tube	NA	NA	Yes	Yes	3‐D reconstruction, TEM	Wilms (1985)
19	*Catananche caerulea*	Compositae/Lactuceae	Young to mature pollen grain (GC to SC formation)	Yes	Yes	Yes	NA	SEM	Barnes and Blackmore (1987)
20	*Brassica oleracea, Z. mays*, and *Triticum aestivum*	Brassicaceae/Poaceae	Mature pollen grain	NA	NA	Yes	NA	Light and epifluorescence DAPI	Matthys‐Rochon et al. (1987)
21	*B. oleracea*	Brassicaceae	Mature pollen grain	NA	NA	Yes	NA	EM	Dumas et al. (1985)
22	*Brassica campestris* and *B. oleracea*	Brassicaceae	Mature pollen grain	NA	NA	Yes	NA	3‐D reconstruction, TEM	McConchie, Russell, et al. (1987)
23	*B. campestris*	Brassicaceae	Mature pollen grain	NA	NA	Yes	NA	3‐D reconstruction quantitative TEM	McConchie et al. (1985)
24	*H. vulgare*	Poaceae	Young to mature pollen grain (GC to SC formation)	No	No	Yes	NA	EM	Charzyńska et al. (1988)
25	*H. vulgare*	Poaceae	Mature pollen (sperm cells)	NA	NA	No	NA	3‐D reconstruction and quantitative TEM	Mogensen and Rusche (1985)
26	*H. vulgare*	Poaceae	Post‐pollination analysis of sperm cells in pollen tube	NA	NA	NA	Yes	3‐D reconstruction quantitative TEM	Mogensen and Wagner (1987)
27	*H. vulgare*	Poaceae	Uninucleate microspore to mature sperm cells at anthesis	No	No	No	NA	EM	Cass and Karas (1975)
28	*H. vulgare*	Poaceae	Mature pollen (sperm cells)	NA	No	No	NA	Light and EM	Cass (1973)
29	*A. thaliana*	Brassicaceae	Mature pollen grain and pollen tube germination	NA	NA	Yes	Yes	Light and epifluorescence microscopy DAPI, CLSM	Lalanne and Twell (2002)
30	*A. thaliana*	Brassicaceae	Microsporogenesis and microgametogenesis	No	No	No	NA	Light and TEM	Owen and Makaroff (1995)
31	*A. thaliana*	Brassicaceae	Mature pollen and sperm cells movement in pollen tube	NA	NA	Yes	Yes	Fluorescence microscopy and CLSM	Ge et al. (2011)
32	*A. thaliana*	Brassicaceae	Mature pollen and sperm cells inside pollen tube	NA	NA	Yes	Yes	SEM and epifluorescence DAPI	Xie et al. (2010)
33	*A. thaliana*	Brassicaceae	Microspore to mature pollen at anthesis	No	No	No	No	EM	Kuang and Musgrave (1996)
34	*A. thaliana*	Brassicaceae	Sperm cells movement in pollen tube	NA	NA	NA	Yes	DIC, DAPI, light, fluorescence, and TEM	Lennon et al. (2000)
35	*A. thaliana*	Brassicaceae	Mature pollen and sperm cells inside pollen tube	NA	NA	Yes	Yes	Live cell fluorescence microscopy, TIRP, DAPI, and CLSM	Vogler et al. (2015)
36	*A. thaliana*	Brassicaceae	Late microspore to mature pollen with two sperm cells	No	No	No	No	Light and TEM	Zając (1997)
37	*A. thaliana*	Brassicaceae	SCs and VN in pollen tube	NA	NA	NA	No	CLSM, DAPI, light, and TEM	Derksen et al. (2002)
38	*A. thaliana*	Brassicaceae	In vitro/semi in vitro pollen germination	NA	NA	Yes	Yes	Fluorescence microscopy and CLSM	Zhou and Meier (2014)
39	*A. thaliana*	Brassicaceae	Mature pollen	NA	NA	Yes	NA	Light and epifluorescence DAPI	Reňák et al. (2012)
40	*A. thaliana*	Brassicaceae	Early to mature pollen with two sperm cells	No	No	No	No	EM	Yamamoto et al. (2003)
41	*A. thaliana*	Brassicaceae	Pollen in vitro germination and sperm cells analysis in pollen tube	NA	NA	NA	Yes	Light and fluorescence microscopy	Sprunck et al. (2014)
42	*A. thaliana*	Brassicaceae	Sperm cells in vivo analysis pollen tube	NA	NA	NA	Yes	Time‐lapse imaging of fluorescence markers	Hamamura et al. (2011)
43	*A. thaliana*	Brassicaceae	SC in vitro analysis in mature pollen and pollen tube	NA	NA	Yes	Yes	Fluorescence microscopy/CLSM Hoechst 33342, GUS	Goto et al. (2020)
44	*A. thaliana*	Brassicaceae	SC in vitro analysis in mature pollen and pollen tube	NA	NA	Yes	Yes	Fluorescence microscopy/CLSM	Zhou and Meier (2014)
45	*Dalzellia zeylanica*	Podostemaceae	Sporogenesis, gametogenesis, and fertilization	No	No	Yes	NA	CLSM, DAPI, light, and SEM	Sehgal et al. (2011)
46	*Sambucus nigra*	Brassicaceae	Mature pollen grain	NA	NA	Yes	NA	TEM, SEM, and fluorescent optical microscopy	Muccifora et al. (2003)
47	*Lampranthus* and *Delosperma cooperi*	Aizoaceae	Sperm cells in mature pollen and pollen tube	NA	NA	Yes	Yes	Stereo, fluorescence microscopy (DAPI) and flow cytometry	Braun and Winkelmann (2016)
48	*Carthamus tinctorius*	Asteraceae	Early to mature pollen with two sperm cells	NA	NA	Yes	Yes	Light and fluorescence microscopy toluidine blue (TBO), DAPI	Yeung et al. (2011)

Abbreviations: BCP, bicellular pollen; TCP, tricellular pollen; GC generative cell; SC sperm cell; DIC, differential interference contrast; LM, light microscopy; EM, electron microscopy; TEM, transmission electron microscopy; CLSM, confocal laser scanning microscopy; IF, immunofluorescence; α, alpha; FM, fluorescence microscopy; LM, light microscopy; FCM, flow cytometry; DAPI, 4′,6‐diamidino‐2‐phenylindole; MT, microtubules; MF, microfilament; YFP, yellow fluorescent protein; TBO, toluidine blue; DiOC6, 3,3‐dihexyloxacarbocyanine Iodide; FISH, fluorescence in situ hybridization.

In Arabidopsis, the physical connection between the SC pair and VC nucleus to form the MGU has been documented previously in mature pollen via light, epifluorescence, live cell imaging of marker lines, and confocal laser scanning microscopy (CLSM) microscopic methods (Ge et al., 2011; Goto et al., 2020; Hamamura et al., 2011; Lalanne & Twell, 2002; Motomura et al., 2021; Reňák et al., 2012; Vogler et al., 2015; Xie et al., 2010; Zhou & Meier, 2014). However, there is limited information about the developmental dynamics of MGU assembly (Lalanne & Twell, 2002), and it remains unknown whether this association also exists at different bicellular pollen stages in Arabidopsis. The physical contact between the male germline cells and the vegetative nucleus (VN) via the CP establishes cell‐to‐cell communication, which might play an important role in the delivery of substances and/or transport of an intact MGU through the pollen tube for the process of double fertilization (McCue et al., 2011).

It was proposed that the physical association of SC (SC1) with the VN facilitates the transfer of material or information between these two structures (Mogensen, 1992). Furthermore, the transport of substances may be directly from the VN to the SC through CP (as there is a membrane continuity between the vegetative nuclear envelope and plasma membrane surrounding GC/SC) or indirectly from the VC cytoplasm via membrane transport. The nuclear pore density is much greater where the GC/SC is associated with the VC nucleus in *Medicago sativa* (Shi et al., 1991). The MGU association between SC and VN provides an increased surface area for transport and interaction (Yu et al., 1992). Therefore, the increased surface area will need more energy for trafficking at the interface, and it has been reported that high ATPase hydrolysis occurs in the pollen of *Hippeastrum vitatum* and *Clivia miniata* (Pei‐hua, 1988). The unidirectional transfer from the VC nucleus to SCs is suggested based on findings that an artificial microRNA expressed from the VC nucleus can reduce the expression of a target green fluorescent protein (GFP) reporter transcript in Arabidopsis SCs (Slotkin et al., 2009), while it has been reviewed that microRNA acts autonomously in plant body (Voinnet, 2005). The molecular mechanism has also been determined where the VC regulates transcriptional reactivation of transposable elements (TEs) and those TE transcripts degrade into small interfering RNAs (siRNAs), which resulted in silencing in SCs (Martinez et al., 2016). The movement of MGU in the pollen tube is regulated by the presence of WIPs (WPP domain‐interacting proteins) and WITs (WPP domain‐interacting tail‐anchored proteins) proteins (Zhou & Meier, 2014), while the nuclear laminal protein, KAKU4 deficiency transforms the folded VN to a spherical form, which has resulted in low fertilization in *A. thaliana* (Goto et al., 2020).

There are convincing pieces of evidence about the MGU as a structural unit, and growing indication for its role as a conduit for cell‐to‐cell communication; however, the genetic and molecular mechanisms of this association remain largely obscure, which has been previously claimed to be regulated by *GUM* (Lalanne & Twell, 2002). Here, we describe the development of the MGU from its inception in early bicellular pollen and examine its dynamic organization in developing pollen of wild‐type Arabidopsis as well as in several independent regulatory classes of bicellular pollen mutants. In addition to these mutants, the allelic *germ unit malformed (gum1–1/gum1–2*) mutants were revisited that were known to regulate the MGU association (Lalanne & Twell, 2002) by determining the presence of MGU through the introduction of double fluorescent markers. Therefore, we decided to mark the male germline (GC/SC) and vegetative nuclear membrane with fluorescent markers for the cellular morphology and confirm the presence of MGU association through CP. The MGU mutants (i.e., *gum*/*mud*) were identified in the EMS mutagenized wild‐type *A. thaliana* pollen (Lalanne & Twell, 2002). In the *mud* (MGU displaced) mutant, the intact MGU is displaced toward the pollen grain wall, while in the *gum* mutant, the physical contact between the SC and VN (VN) appears to be lost at late tricellular pollen stages. It was proposed that *MUD* potentially regulates the position of the MGU and *GUM* encode proteins to keep the SC and VN linked and/or close to each other (Lalanne & Twell, 2002).

The *duo1* and *duo2* mutants were found in an EMS‐mutagenized DAPI screen in Arabidopsis pollen, where the *duo2* mutant GC enters mitosis but arrests at prometaphase, highlighting the role of DUO2 in mitotic progression and mutant GC in *duo1* completes the S‐phase but fails to enter mitosis (Durbarry et al., 2005). DUO POLLEN 1 (DUO1) was the first transcription factor shown to be expressed specifically in the GCs (Rotman et al., 2005), and its transcript level peaks during male gametogenesis before GC division (Brownfield et al., 2009). Similarly, the GC division is regulated by DUO2 independently of DUO1, based on the HTR10 (DUO1 target) expression in the *duo2* mutant germline (Figure S12) as the gene has not been characterized and the molecular mechanism of mutation is unknown. The DUO POLLEN 3 (DUO3) regulates GC division because mutant GC enters the S‐phase but fails to divide, while coordinates with DUO1 for the expression of GEX2 and GCS1 (HAP2) in the germline (Brownfield et al., 2009; Brownfield & Twell, 2009). Furthermore, it has been proposed that both DUO1 and DUO3 activate an overlapping set of germline differentiation genes in the GC just after its formation, where DUO1 promotes GC entry into mitosis through activation of CYCB1;1 but DUO3 performs the same function independently (Berger & Twell, 2011). The DUO POLLEN 1 ACTIVATED ZINC FINGER 1/2 (DAZ1/DAZ2‐redundant) proteins belong to the C_2_H_2_–type zinc finger protein (ZFP) family genes (Takatsuji, 1998), which have an important role in the G2‐M transition of the GC division and mitotic cyclins accumulation (Borg et al., 2014).

It has been shown that the F‐box like‐17 protein forms a complex with SKP1‐like protein 11 (ASK11), which degrades the Kip‐related protein 6 (KRP6) and KRP7 in the male germline and regulates GC progress through PMII during cell cycle, while mutation of an F‐box protein (FBL17) results in bicellular pollen mutant known as *fbl17* (Kim et al., 2008; Gusti et al., 2009). The plant A‐type cyclin‐dependent kinase (CDKA) is a homolog of the animal cell division cycle protein 2 (CDC2) and plays an important role in the cell cycle. It has been demonstrated that CDKA;1 is required for GC entry into the cell cycle and both G_1_/S and G_2_/M phase transition are under its regulatory control, while loss‐of‐function mutation in CDKA;1 results in bicellular pollen containing a single sperm‐like cell and a VC (Iwakawa et al., 2006).

To confirm the presence of physical contact between SC and VN in the *gum* allelic mutants (*gum1–1*/*gum1–2*) and other bicellular pollen mutants (*duo1*, *duo2, duo3, daz1daz2, cdka;1, fbl17*), the double homozygous marker lines of mutants need to be examined developmentally. To achieve this, we introduced male germline (GC/SC) plasma membrane (TET11‐GFP/DUO1:TET11‐GFP), cytoplasm (HTR10:GFP‐TUA6), and vegetative nuclear membrane (LAT52:RanGAP‐tdTomato) fluorescent markers in the wildtype and different mutants. The application of these molecular tools was very helpful in marking the narrow CP and its association with the nuclear envelope.

## 2 MATERIALS AND METHODS

### 2.1 Plant materials and growth conditions

*A. thaliana* plants were grown in growth rooms on Levington F2S soil (Compost: Sand: Vermiculite, Scotts‐UK) at 22 °C with a 16‐h‐light and 8‐h‐dark cycle or with 24 h light (120 to 140 μ mol/m^2^/s) with 50%–60% relative humidity.

Details of the different mutant alleles of the genes that were analyzed are given in Table S3. These mutant alleles include *duo1–2* (Rotman et al., 2005), *duo1–4* (Borg et al., 2014), *duo2* (Durbarry et al., 2005), *duo3* (Brownfield et al., 2009), *daz1–1 daz2–1* (Scholl et al., 2000), *cdka;1* (Iwakawa et al., 2006; Nowack et al., 2006), *fbl17* (Kim et al., 2008), and *gum1–1*/*gum1–2* (Lalanne & Twell, 2002) (Table S3). Double homozygous marker line seeds of *gum1–1*/*gum1–2* and other mutants (HTR10:GFP‐TUA6 × DUO3:H2B‐tdTomato; HTR10:GFP‐TUA6 × LAT52:RanGAP‐tdTomato) were generated either by transformation or crosses (Table S2). A range of previously described markers and transgenic lines were used (Table S1). The different markers transformed into different mutants were either generated at Twell Lab (HTR10:H2B‐RFP, HTR10:TUA6‐GFP, HTR10:H2B:GFP) or crossed into these mutants (wt TET11‐GFP) or new markers were produced through Multisite Gateway® recombination (DUO1:TET11‐tdTomato/GFP, LAT52:RanGAP‐GFP/tdTomato, DUO3:H2B‐tdTomato) (Table S6‐S7). Further information about gateway cloning is available at http://tools.invetrogen.com/content/sfs/manuals/gatewayman.pdf.

### 2.2 Microscopy

To examine developing pollen for bright field analysis and GFP and red fluorescent protein (RFP) marker expression, anthers from each of the four long (medial) stamens of successive flower bud stages were dissected into 5–10 μl of .3 M mannitol on a glass slide using a 25‐gauge hypodermic needle. Flower developmental stages were staged as described (Lalanne & Twell, 2002): +1, first open flower (mature tricellular pollen); −1 and −2, first and second unopened flower buds (immature tricellular pollen); −3, third unopened bud (pollen mitosis II and bicellular pollen); −4, fourth unopened bud (bicellular pollen). The pollen was mounted in .3 M mannitol and examined using a Nikon Eclipse‐80i microscope. Bright‐field and differential interference contrast (DIC) images were captured with a Nikon‐D100 (Model MH‐18, Japan), and fluorescence images were captured with a HAMAMATSU ORCA‐ER (Model C4742–95, Japan) using the NIS‐Elements software. For examination of pollen nuclei labeled with DAPI (4′‐6‐diamidino‐2‐phenylindole dihydrochloride), pollen was incubated in .8 μg/ml DAPI as previously described (Brownfield et al., 2009).

For confocal microscopy (CLSM), developing pollen mounted in .3 M mannitol was examined with an Olympus‐FV1000 (USA) microscope and Fluoview viewer software (FV10‐ASW Version 04.00.02.09). Optical stacks were taken with an immersion‐oil UPLSAPO 60x/1.35NA objective. GFP signal was imaged with 488 nm excitation and 500–600 nm detection. RFP was excited at 635 nm with detection of 655–755 nm. Hoechst signal was imaged with 405 nm excitation and an emission of 425–475 nm. Images were taken using the same voxel (volume pixel) resolution (*XY* dimension 85 nm, *Z* dimension 250 nm). CLSM images were transformed into 3‐D reconstructions with the IMARIS software (Bitplane AG, An Oxford Instruments Company Badenerstrasse, Zurich Switzerland).

## 3 RESULTS

### 3.1 Current status of the MGU association in angiosperms

The available published literature survey revealed the existence of MGU association in 49 out of 55 bicellular pollen species studied at the developmental stages (Table 1). The MGU was not detected only in *Helleborus foetidus* (Heslop‐Harrison et al., 1986) and *Haemanthus katherinae* (Sanger & Jackson, 1971). Similarly, the MGU has been reported in all the 46 tricellular pollen species studied at the developmental stages, while only in nine studies (study IDs. 6, 25, 27–28, 30, 33, 36–37, 40) that included those on *A. thaliana* (Derksen et al., 2002; Kuang & Musgrave, 1996; Owen & Makaroff, 1995; Yamamoto et al., 2003), *Zea mays* (Rusche & Mogensen, 1988) and *Hordeum vulgare* (Mogensen & Rusche, 1985), but independent studies have subsequently confirmed the presence of the MGU in all three species (Hamamura et al., 2011; Kliwer & Dresselhaus, 2010; Mogensen & Wagner, 1987; Reňák et al., 2012) (Table 2).

### 3.2 The MGU association at bicellular pollen stages

**FIGURE 1:**
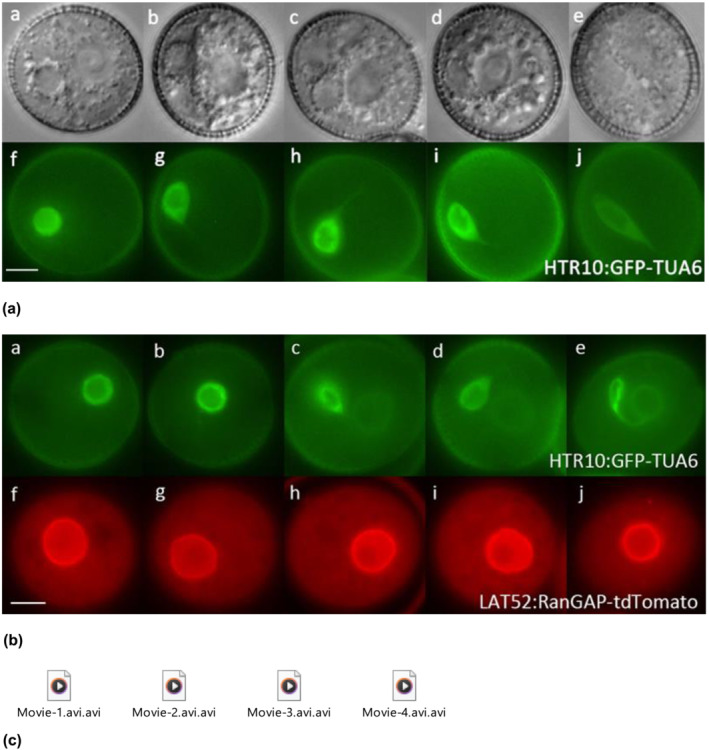
Formation of the MGU in developing bicellular pollen of wild‐type Arabidopsis. (a) Panels a–e show DIC and f–j the corresponding GFP signal from GFP‐TUA6 (germline cytoplasm) images in early bicellular (a, f) to late bicellular (e, j) pollen marker lines (developmental analysis of eight‐independent marker lines and more than 70 pollen grains examined at each independent bud stage). The generative cell initially has a round profile (a, f) and develops a fine thread‐like cytoplasmic projection (g‐j) that seems linked to the vegetative cell nucleus at all stages (b‐e). (b) Panels a–e show the GFP signal from GFP‐TUA6 (germline cytoplasm) and f–j the corresponding RFP signal from RanGAP‐tdTomato (vegetative cell nuclear envelope) in early bicellular (a, b) to late bicellular (e, j) pollen of double marker lines (six‐independent homozygous marker lines analyzed, while the male germ unit association reported in 100% [90 pollen] pollen grains examined at each bud stage). The generative cell is round in profile (a, b) and then develops a fine thread‐like cytoplasmic projection linked to the vegetative cell nuclear envelope at subsequent stages (c–e). Each image represents an independent pollen stage arranged from early to late pollen stages (left to right). Scale bar = 5 μm. (c) Movies. Three‐dimensional reconstruction of the male germ unit in wild‐type at bicellular pollen stages. DIC, differential interference contrast; GFP, green fluorescent protein; MGU, male germ unit; RFP, red fluorescent protein.

In the current in vivo developmental analysis, at the early bicellular pollen stage just after the detachment of the GC from the pollen wall, the GC remains close to the VC nucleus in the majority of pollen grains (Figure 1[a,b]; Figure S1‐S3a). The GC is round in profile and has a fine thread‐like CP (Figure 1(b), Movie S1; Figure S1‐S3), which extends toward the VC nucleus and is physically associated with the vegetative nuclear membrane. We used various fluorescent markers to examine during the in vivo developmental analysis using this association between the GC CP and the VC nucleus is observed at early, mid, and late bicellular bud stages (Early MGU) and the analysis demonstrated that the GC is physically linked with VC membrane through CP (Figure 1(a,b], Movie S2; Figure S1‐S3). The length of the CP increases with GC body morphogenesis, and an elongated CP has been observed at the late bicellular stage (Figure 1(b), Movies S3 and S4; Figure S2‐S3). The physical association between dividing GC and vegetative nuclear membrane appears to remain intact based on the double fluorescent markers analysis (HTR10:GFP‐TUA6 × DUO3:H2B‐tdTomato; HTR10:GFP‐TUA6 × LAT52:RanGAP‐tdTomato). The MGU association has been observed using different sets of fluorescent markers, and the same set of data has been obtained at all these bicellular bud stages (Figure 1(a,b); Figure S1‐S3; Movies S1–S4). Multiple and different sets of fluorescent markers were used in this study to exclude any artifact and reconfirm the MGU association between GC/SC (TET11‐GFP/HTR10:GFP‐TUA6/DUO1‐TET11‐tdTomato) and VN (LAT52:RanGAP‐GFP/DUO3:H2B‐tdTomato). To visualize the MGU association, it was mandatory to label the male germline (GC/SC) and VN (DUO3:H2B‐tdTomato) or vegetative nuclear membrane (LAT52:RanGAP‐GFP) with contrast fluorescent tags. Furthermore, HTR10 is a target of DUO1; therefore, the fluorescent marker (HTR10:GFP‐TUA6) has no expression in multiple mutant alleles of DUO1 (*duo1–1*, *duo1–2*, *duo1–3*, *duo1–4*), and we have explored alternate fluorescent markers (TET11‐GFP/DUO1‐TET11‐tdTomato).

### 3.3 The MGU association at tricellular pollen stages

**FIGURE 2:**
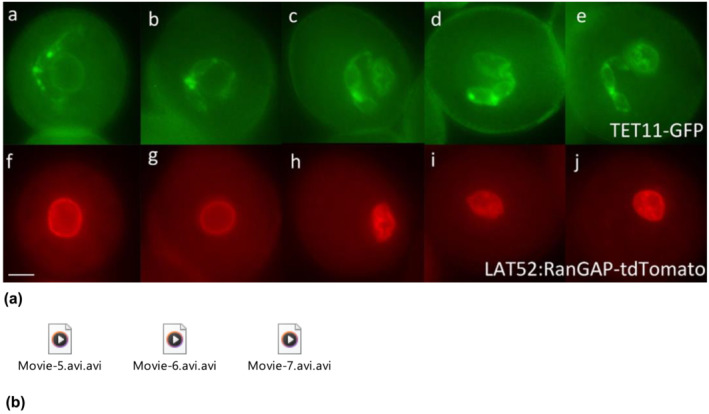
Male germ unit in developing tricellular pollen of wild‐type Arabidopsis. (a) Panels a–e show the GFP signal from TET11‐GFP (germline plasma membrane) and f–j the corresponding RFP signal from RanGAP‐tdTomtato (vegetative cell nuclear envelope) in early tricellular (a, f) to late tricellular (e, j) pollen of a double marker line. One of the sperm cell pairs is always linked to the vegetative cell nuclear envelope through its cytoplasmic projection (six‐independent homozygous marker lines analyzed, where the male germ unit association has been observed in 100% [150–200 spore observed] pollen analyzed at these different tricellular pollen stages). Each image represents an independent pollen stage arranged from early to late pollen stages (left to right). Scale bar = 5 μm. (b) Movies. Three‐dimensional reconstruction of the male germ unit in wild‐type at different tricellular pollen stages. GFP, green fluorescent protein; RFP, red fluorescent protein.

**FIGURE 3:**
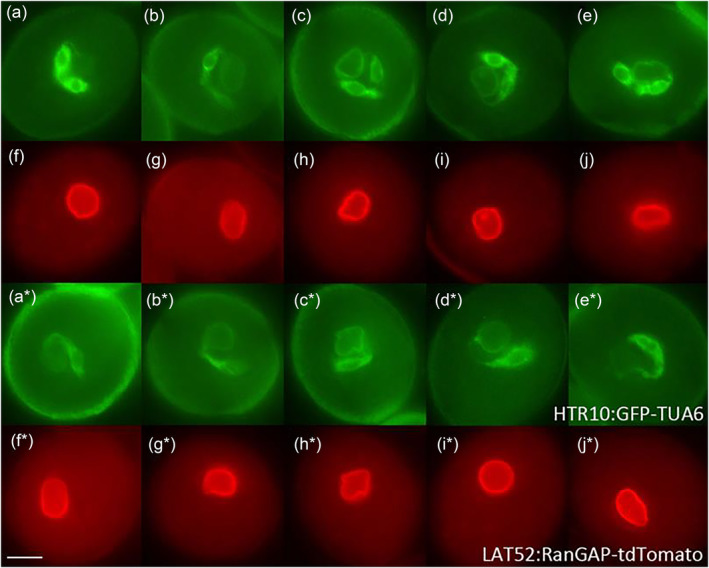
Male germ unit in the undivided generative cell of *fbl17* mutant pollen at tricellular pollen stages. Panels a–e show the GFP signal from GFP‐TUA6 (germline cytoplasm) and f–j the corresponding RFP signal from RanGAP‐tdTomato (vegetative cell nuclear envelope) in early tricellular (a, f) to late tricellular (e, j) stage pollen of a double marker line. In the wild type (a–e) one sperm cell is always linked to the vegetative cell nuclear envelope through its cytoplasmic projection (CP), while the undivided generative cell of *fbl17* pollen is also connected to the vegetative cell nuclear envelope via a CP (a*–e*) (five‐independent homozygous mutant marker lines analyzed and the male germ unit has been observed in 100% [150–200] pollen grains of wild and mutant examined at these different tricellular pollen stages). Each image represents an independent pollen stage arranged from early to late pollen stages (left to right). Scale bar = 5 μm. GFP, green fluorescent protein; RFP, red fluorescent protein.

The GC divides symmetrically by pollen mitosis‐II (PMII) into two SCs (SCs) of equal sizes. One of the SCs has a long CP, and the other has a small (87%) or two CPs of equal length (13%) in *A. thaliana* (Rauf et al., 2023). One of the SCs with long extension is physically linked with the vegetative nuclear membrane and forms the MGU at tricellular pollen stages (Late MGU) (Figure 2, Movies S5–S7). The MGU association has been observed at early, mid, and late tricellular pollen stages between the SC and vegetative nuclear membrane based on the in vivo analysis of the different sets of fluorescent markers in wild type *A. thaliana* (Figures 2, 3/6, Movies S5–S7; Figure S4‐S5).

Further, the developmental analysis of different bicellular pollen mutants (*duo1–4*/*duo1–2*/*fbl17*/*cdka;1*/*daz1daz2*/*duo2*/*duo3*) demonstrated that the MGU association exists between the mutant GC and vegetative nuclear membrane developmentally (Figure 3[a–e]; Figure S8‐S12/S15). We have analyzed and presented data from five independent homozygous marker individuals for each mutant. Our analysis shows that DUO1, FBL17, CDKA;1, DAZ1 DAZ2, DUO2, and DUO3 have no potential role in the MGU association, while their role in the development of the CP was excluded previously (Rauf et al., 2023).

### 3.4 The MGU association is independently regulated of *GUM*

**FIGURE 4:**
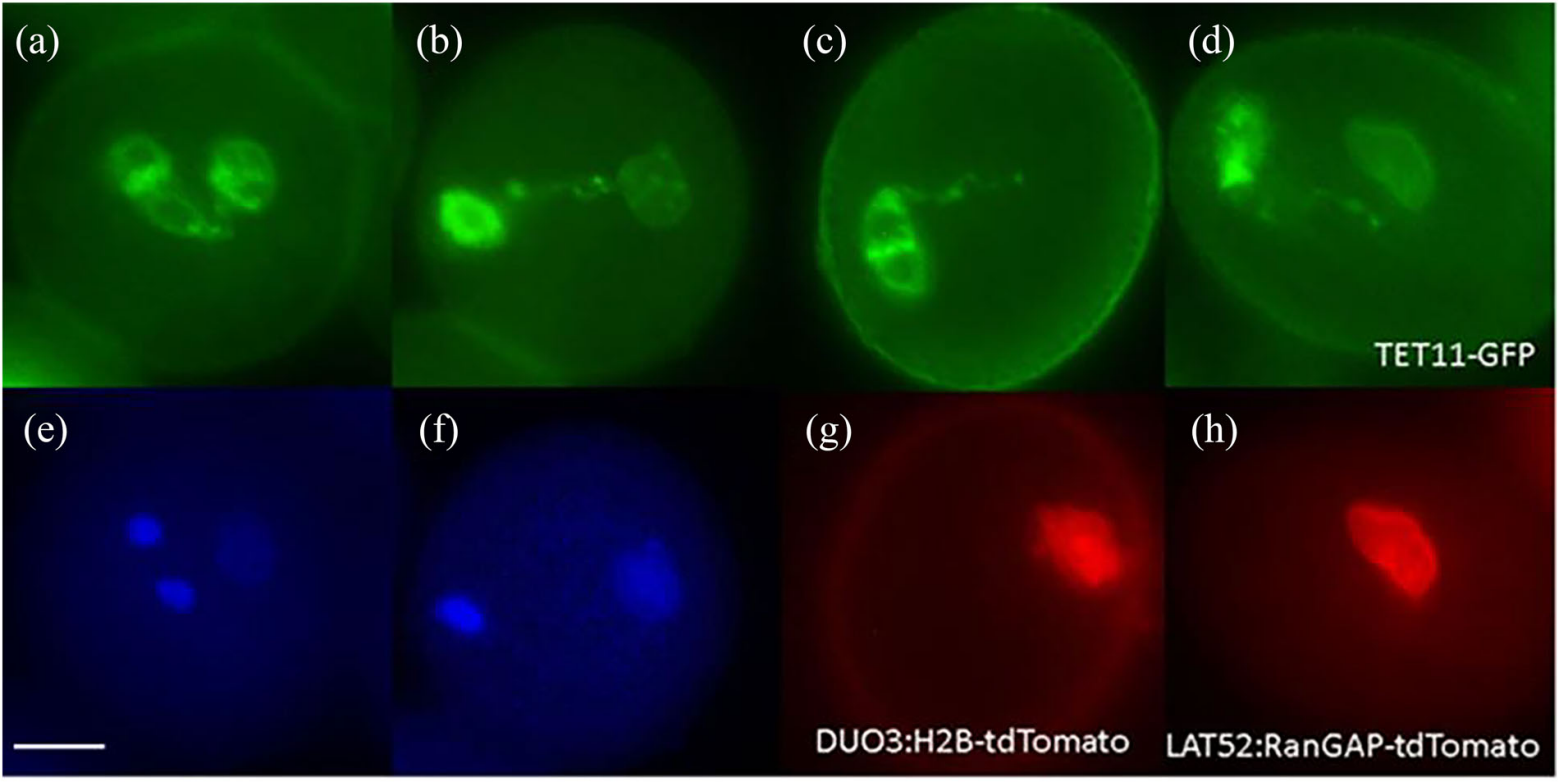
Micrographs of wild type and *gum* showing the position of the male germ unit components. The two sperm nuclei are in the center close to the vegetative nucleus in wild type (a and e), while in *gum* the association between two sperm nuclei and the vegetative nucleus appears to be lost in DAPI (f) but the fluorescent images reveal physical contact (b–d and g–h). Scale bar = 5 μm.

Out of the two MGU mutants (*mud* and *gum*) in *A. thaliana*, it was proposed that in *gum* mutant, physical contact between the SC and VN appears to be lost, and therefore, *GUM* might encode proteins to keep the SC and VN linked and/or close to each other (Figure 4) and a potential candidate gene involved in the MGU association (Lalanne & Twell, 2002). We did not present developmental data of *mud* (MGU displaced) mutant since the organization was not affected but instead, the whole MGU was displaced from the centre to the periphery (Lalanne & Twell, 2002). Therefore, in vivo, fluorescence microscopic analysis of *gum* mutants (*gum1–1/1–2*) double homozygous marker lines, i.e., TET11‐GFP × DUO3:H2B‐tdTomato and TET11‐GFP × LAT52:RanGAP‐tdTomato, may help to elucidate the organization and assembly of the MGU as well as the molecular mechanisms.

**FIGURE 5:**
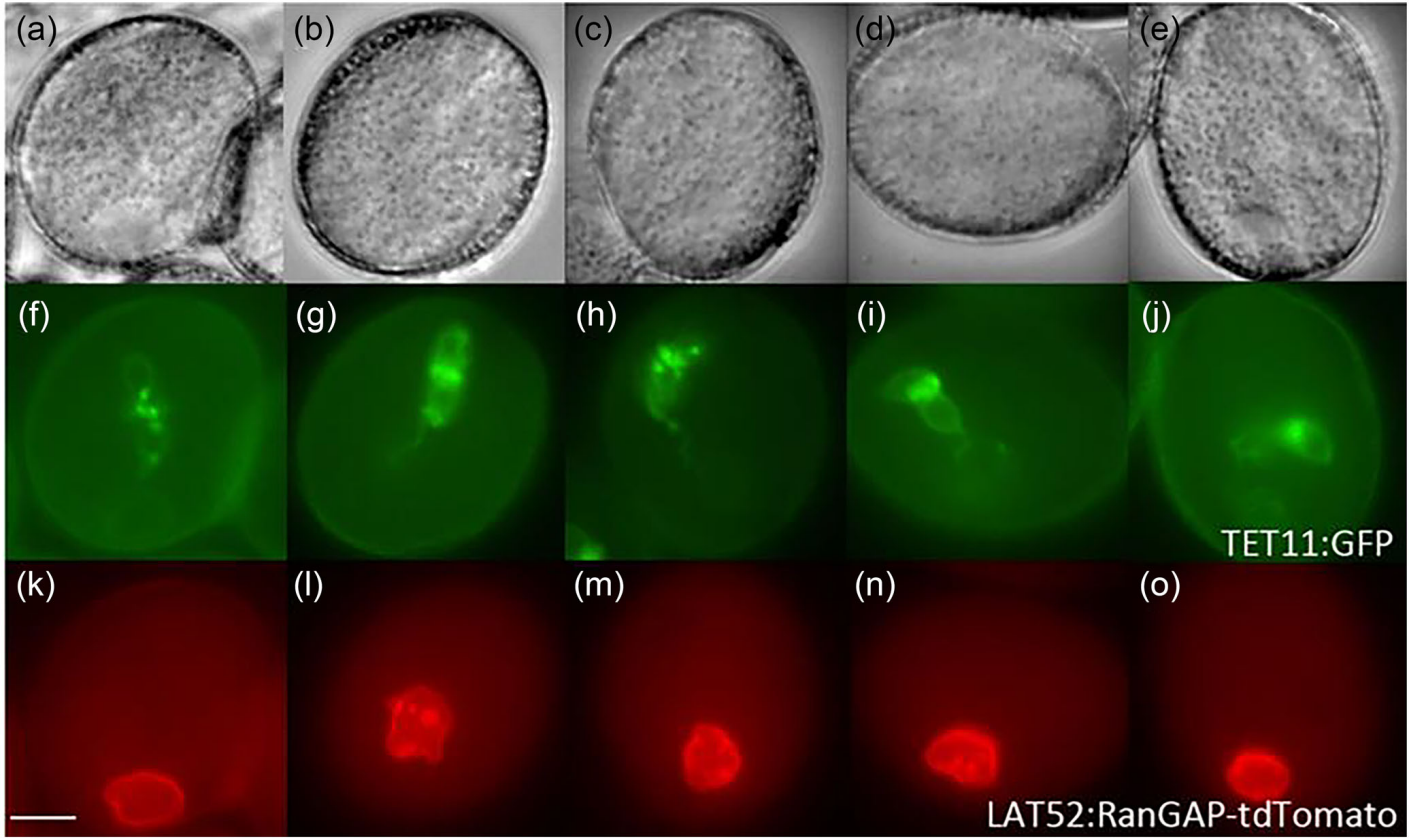
Intact male germ unit in *gum1–2* pollen at tricellular pollen stages. Panels a–e show DIC, f–j the GFP signal from TET11‐GFP (germline plasma membrane), and k–o the corresponding RFP signal from RanGAP‐tdTomato (vegetative cell nuclear envelope) in early tricellular (a, f, k) to late tricellular (e, j, o) pollen of a double marker line. One of the sperm cell pairs always appears linked to the vegetative cell nuclear envelope through its cytoplasmic projection even though the vegetative cell nucleus is located peripherally rather than centrally (lower panels, k‐o) (five‐independent homozygous marker lines analyzed developmentally for the presence of physical association in 100–140 pollen grains at different bud stages). Each image represents an independent pollen stage arranged from early to late bicellular pollen stages (left to right). Scale bar = 5 μm. GFP, green fluorescent protein; RFP, red fluorescent protein.

The developmental analysis of two different sets of homozygous double marker lines of *gum1–2* ^*−/−*^ *TET11‐GFP×DUO3:H2B‐tdTomato* and *gum1–2* ^*−/−*^ *TET11‐GFP×LAT52:RanGAP‐tdTomato* showed that the germline has a physical association with the vegetative nuclear envelope through CP (Figure 5[f–j]; S13), irrespective of the SCs and VN located far from each other inside pollen grain, which was previously presumed that the MGU association might be lost in *gum1–1*/*gum1–2* (Figure 4) in the DAPI analysis (Lalanne & Twell, 2002).

The physical association between germline and vegetative nuclear envelope has been observed through CP at various bud stages in the five independent homozygous backed‐crossed‐6 (BC‐6) *gum1–2* (100%) mutant lines (Table S4). Homozygous mutant lines of *gum* were analyzed at the bicellular and tricellular pollen stages. It has been demonstrated that *GUM* affects the MGU association only at tricellular pollen stages (Lalanne & Twell, 2002); therefore, developmental data of the bicellular pollen stages are not shown. This demonstrates that *GUM* may regulate the cytoskeleton or cellular network which keeps the two components of the MGU close to each other but not the organization and physical assembly of the MGU.

### 3.5 Presence of physical contact between a SC and VN in *gum 1–1*

The MGU association has also been analyzed in the second allele of *GUM* (*gum1–1* ^*−/−*^) by introducing different fluorescent markers to label both germline and vegetative nuclei as mentioned in the case of *gum1–2*. The developmental analysis of five independent homozygous backed‐crossed‐6 (BC‐6) double marker lines of *gum1–1* ^*−/−*^ (*TET11‐GFP × LAT52:RanGAP‐tdTomato*; *DUO1:TET11‐tdTomato × LAT52:RanGAP‐GFP*; *TET11‐GFP × DUO3:H2B‐tdTomato*) determines that one SC of pair is physically linked and the MGU association has not been lost in *gum1–1* ^*−/−*^, irrespective that the two SCs are situated far from the VN and the MGU association remains intact (Figure S14). The physical association between germline and vegetative nuclear envelope has been observed through CP at various bud stages in *gum1–1* ^*−/−*^ (100%) (Table S5). This determines that the physical association between germline and VN in the form of MGU is completely independent of the proposed *GUM* regulator and genetic and molecular mechanisms need to be explored.

## 4 DISCUSSION

Morphogenesis and elongation of the GC body and its CP are unique features of angiosperms. However, both developmental processes are regulated independently based on the analysis of multiple mutant alleles of *DUO1* (Rauf et al., 2023). In that paper, we have shown that the male gametophyte bicellular pollen mutants (*duo1–1*, *duo1–2*, *duo1–3*, *duo1–4*, *cdka;1, fbl17*)/(*daz1 daz2, duo2, duo3*‐Rauf, 2017) form the CP, while here we confirmed the presence of MGU association, which exclude their potential role in this assemblage. The GC possesses CP in wild type (Figure 1(a,b)), different bicellular pollen mutants (Figure 3[a*–e*]; Figure S8‐S12/S15), and *gum* mutants (*gum1–1*/*gum1–2*) (Figures 4 and 5; Figure S13‐S14) in *A. thaliana*.

The important role of male germline CP has been reviewed (Dumas et al., 1998; McCue et al., 2011; Mogensen, 1992). The physical linkage of one SC of the pair with the VN, via a CP was reported in cotton pollen grain (Jensen & Fisher, 1968). This association forms the structural and functional unit of the organization, known as the MGU (Dumas et al., 1985). This physical association between the male germline and the VN has also been observed at different bicellular (Figure 1(a,b), Movie S1–S4) and tricellular (Figure 2, Movies S5–S7) developmental stages in *A. thaliana* and the same in other plant species including *Cymbidium goeringii* (Yu & Russell, 1992), *Tropaeolum majus* (Niu et al., 1999), *Plumbago zeylanica* (Russell & Strout, 2005), and *Z. mays* (Kliwer & Dresselhaus, 2010). The analysis of fluorescently labeled male gametophytes could help to determine the presence of the MGU in the remaining different species of angiosperm. The majority of the angiosperms have MGU association (Tables 1 and 2), while our current developmental analysis has demonstrated the striking importance of in vivo imaging for the confirmation of narrow CP of male germline (GC/SC) and the broad conservation of the persistent MGU contact in all the angiosperms and its important role in successful double fertilization.

While the mechanisms regulating ontogeny of the CP and its role in the establishment of the MGU are unknown, it was hypothesized that the physical association between the SC and VN might be disturbed or lost in the *gum* mutants (*gum1–1*/*gum1–2*) (Lalanne & Twell, 2002; Figure 4). The in vivo developmental analysis of double marker lines of *gum1–1/gum1–2* mutants revealed that the physical association between the SC and VN is sufficient to prevent dissociation, despite separation of the two structures in *gum* (Figures 4 and 5). We have analyzed five independent homozygous backcrossed‐6 (BC6) *gum1–1*/*gum1–2* double fluorescent marker lines, while an intact physical association was observed in the displaced MGU. The pollen development was also analyzed at different bicellular pollen stages in the homozygous backcrossed‐6 individuals, but the *gum* phonotype appeared only at the tricellular pollen stages (Lalanne & Twell, 2002); therefore, data is not shown. Further, it was speculated that bicellular pollen mutants might also show defects in the MGU organization and development of the MGU but an intact association has been observed in pollen of the various mutant alleles including *duo1–2, duo1–4, duo2, duo3, daz1 daz2, fbl17, and cdka;1*, and thus, the process is independently regulated of these bicellular pollen mutants (Figure 3; Figure S8‐S12/S15).

### 4.1 The MGU association is unaffected and independently regulated of GUM in *A. thaliana*

The MGU is the structural and functional unit in the majority of angiosperms (Dumas et al., 1984), and an attempt was made to find the genetic and molecular mechanisms of the MGU association and establishment in *A. thaliana* (Lalanne & Twell, 2002). The in vivo fluorescence microscopic analysis of double marker lines demonstrated an intact MGU in *gum* (Figure 4/S13‐S14), which had been reported might be a potential regulator of the MGU organization and ontogeny in *A. thaliana* due to the apparent loss of physical linkage (Lalanne & Twell, 2002). Therefore, it remains unresolved to determine which genes/factors regulate the establishment of the MGU in *A. thaliana*. The connection between SC and VC nucleus through CP is firm enough that even displacement of the two structures from each other does not cause the dissociation of the linkage in *gum* mutants (Figures 4 and 5). This provides an opportunity to explore and determine the genetic and molecular mechanisms regulating the establishment of the MGU using *A. thaliana*.

### 4.2 Early and late MGU association in *A. thaliana*

In most of the plant species, the MGU association has been observed between SC and VN at anthesis including *P. zeylanica* (Russell, 1984; Russell & Cass, 1981), *Brassica oleracea* (Dumas et al., 1985; McConchie et al., 1985, McConchie, Russell, et al., 1987), *Spinacia oleracea* (Theunis et al., 1991; Wilms, 1985) and *Nicotiana tabacum* (Yu & Russell, 1994) or in pollen tube in *Cyphomandra betacea* (Hu & Yu, 1988), *Hippeastrum vitatum* (Mogensen,  1986b), *N. tabacum* (Palevitz, 1993; Tian et al., 1998, 2001; Yu et al., 1989, 1992; Yu & Russell, 1994), *Petunia hybrida* (Wagner & Mogensen, 1988), *Populus deltoides* (Rougier et al., 1991), *Rhododendron laetum* and *R. macgregoriae* (Taylor et al., 1989), and *Z. mays* (Rusche & Mogensen, 1988).

**FIGURE 6:**
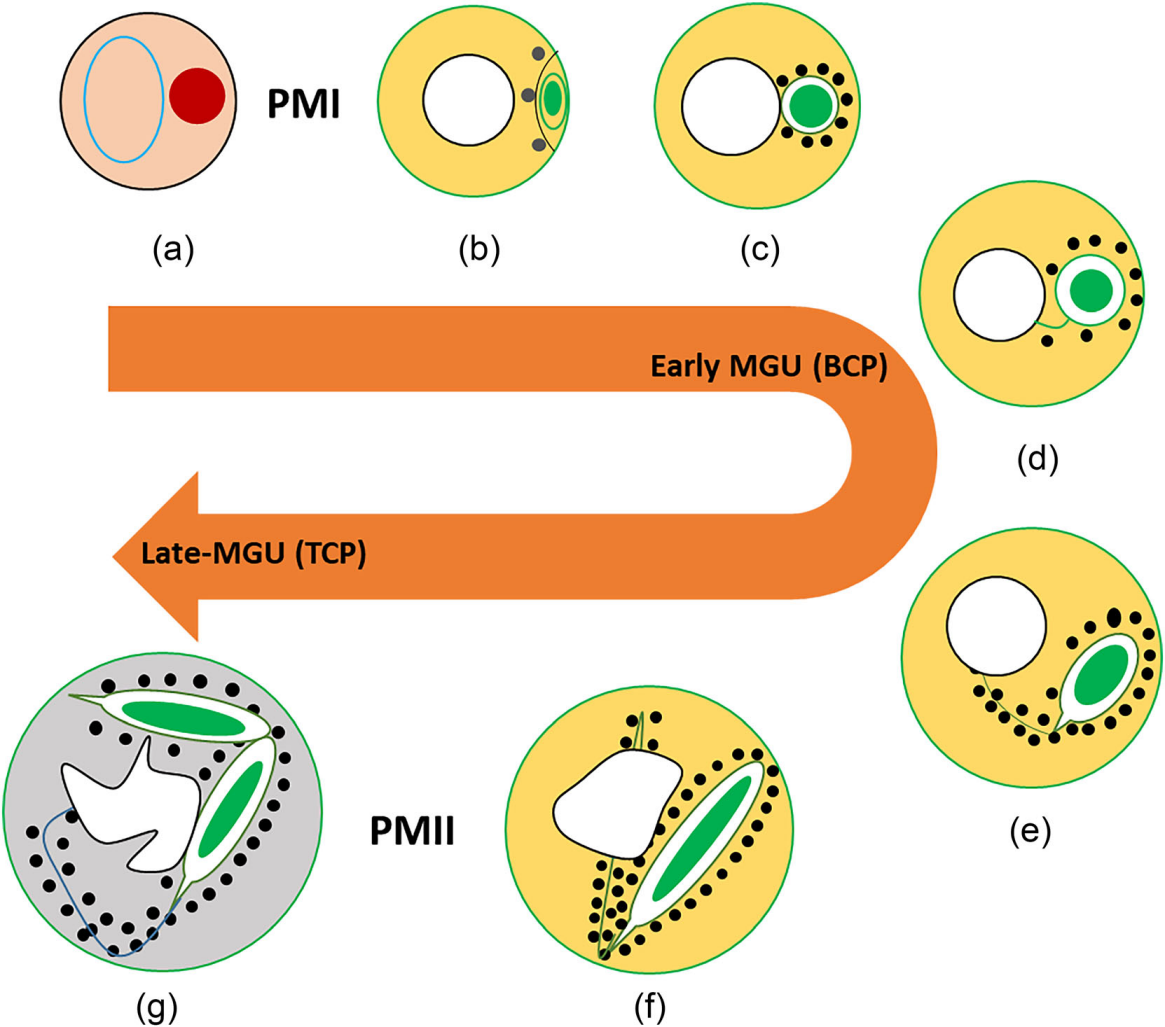
Schematic development of pollen showing MGU association at the bicellular pollen and tricellular pollen stages. The polarized haploid microspore (a) divides asymmetrically (PMI) into bicellular pollen with a lens‐shaped generative cell (GC) attached to the pollen wall (b). The round generative cell is positioned close to the vegetative nucleus (VN), while a physical link through a cytoplasmic projection is established between the generative cell and vegetative nucleus (i.e. early MGU‐c‐d). The elongated generative cell has a long cytoplasmic projection, whereas the latter links with the vegetative nucleus (e–f). Two sperm cells are formed through symmetric division (PMII) and one of the sperm cells has a physical linkage with the vegetative nucleus (late MGU‐g). The black dots represent lipid droplets, which have a high concentration on the surfaces of the plasma membrane (GC/SC). PMI, pollen mitosis‐I; PMII, pollen mitosis‐II.

The MGU association has not been analyzed at early stages in the pollen grain in many plants, and detailed data is limited, for evidence of the GC physical linkage with the VN at bicellular pollen stages (Early MGU) (Kliwer & Dresselhaus, 2010; Palevitz, 1993; Yu & Russell, 1992, 1994). It has also been determined during in vivo developmental analysis that the round GC is situated very close to the VN and linked with the vegetative nuclear membrane through CP and forms the MGU at different bicellular (Early MGU) (Figure 1(a,b), Movies S1–S4) and tricellular (Late MGU) (Figure 2, Movies S5–S7) bud stages in *A. thaliana* (Figure 6), while a recent review proposed the presence of MGU association only at late bicellular and tricellular pollen stages (McCue et al., 2011). Most of the previous developmental analyses have been performed to analyze the MGU association between the GC CP and VN using either immunolocalization of MT or electron microscopy or 3‐D reconstruction. In the present study, various alternate strategies were used to mark the GC CP (cytoplasm/microtubule marker‐HTR10:GFP‐TUA6; plasma membrane marker‐TET11‐GFP/DUO1:TET11‐tdTomato), the VC nuclear membrane (LAT52:RanGAP‐tdTomato), and both GC and VC nuclei (DUO3:H2B‐tdTomato) (Table S2). The live pollen analysis using both light and fluorescence microscopy demonstrates that the round GC at early stages positions very close to the vegetative nuclear membrane and appears to be physically linked with the VC nuclear membrane through its CP to form the MGU (Figure 1(a,b), Movie S1). In *Cymbidium goeringii*, some of the GCs lose their CPs (Yu & Russell, 1992), or in *N. tabacum*, the GC extensions are fully or partially withdrawn, and the MGU dissociates at GC division at metaphase and the association is re‐established at tricellular pollen stage (Kliwer & Dresselhaus, 2010; Niu et al., 1999; Palevitz, 1993; Yu & Russell, 1994). In contrast, no such loss or shortening of CPs nor dissociation of the MGU was observed at any stage of development in *A. thaliana* (Figure 1(b); Figure S7; Movies S2–S4). Generally, it is presumed that the male germline body (GC/SC) elongation might be necessary for the association and establishment of the MGU. Our analysis has well established that only CP elongation and development are required for the MGU association since if the GC body undergoes complete (*fbl17/cdka;1*), partial (*duo2/duo3/daz1daz2)*, or no (*duo1*) morphogenesis in these different bicellular pollen mutants, an intact MGU can be formed (Figure 2; Figure S8–S10/S15). This further strengthens the discovery that the ontogeny of CP and GC elongation is independently regulated (Rauf et al., 2023).

### 4.3 The newly formed SCs are physically linked in Arabidopsis

The twin SCs are physically linked with each other, while one of them is physically associated with the VC nucleus via a CP in most tricellular pollen species and bicellular pollen species as mentioned earlier (Tables 1 and 2). We observed a similar pattern at all developmental stages in the male gametophyte developmental analysis in *A. thaliana*. In contrast, the two SCs separate from each other and lose their CPs at tricellular pollen stages inside the pollen grain in *H. vulgare* (Cass, 1973; Cass & Karas, 1975; Mogensen & Rusche, 1985). The light microscopic examination showed that both sperm nuclei appear far from each other (1.6 μm) as well as distant from the VN (1.6 μm) inside pollen tube in barley (Pope, 1937), while the dissociation between SCs and VN has also been reported in Triticale (Schröder, 1983), *H. foetidus* (Heslop‐Harrison et al., 1986), and *Alopecurus pratensis* (Heslop‐Harrison & Heslop‐Harrison, 1984).

Except for a few plant species like *H. foetidus* that lack the MGU (Heslop‐Harrison et al., 1986), where the reported GC appears to have tapering ends (CPs) and the revisiting might confirm the presence of MGU. The MGU is observed in mature pollen at anthesis (Charzyńska et al., 1988) and during the passage of SCs within pollen tubes in *H. vulgare* (Mogensen & Wagner, 1987), which has been previously reported to show deviation from the rest of Angiosperms during pollen development (Cass, 1973; Cass & Karas, 1975; Mogensen & Rusche, 1985). However, further detailed observations of live cells including MT‐labelling are required to confirm the MGU in these species because the fine CP is difficult to visualize without labelling either with fluorescent markers or anti‐MT or 3‐D reconstruction. The 3‐D reconstruction of the MGU in different plant species has shown that the GC is physically associated with a VN through CPs (Theunis et al., 1985; Mogensen, 1986; McConchie, Hough, & Knox, 1987; Zhu et al., 1990; Yu & Russell, 1992; Palevitz, 1993; Yu & Russell, 1994; Russell & Strout, 2005, Hirano & Hoshino, 2010; Reviewed in McCue et al., 2011) and the same was observed in Arabidopsis 3‐D analysis (Movies 1–7). Further, in vivo analysis of fluorescent marker lines in tobacco and maize also revealed that the GC CP is linked with the VC nucleus (Kliwer & Dresselhaus, 2010; Oh et al., 2010), while the same was observed in the developmental analysis of *A. thaliana* (Figures 1, 2, 3, 4, 5).

## 5 CONCLUSIONS

GCs and SCs develop fine CPs, and thus, all angiosperms potentially have the MGU association during male gametophyte development based on our analysis. Therefore, it seems that there might be an intact association between male germline cells and VN throughout pollen development in flowering plants based on the in vivo developmental analysis of multiple fluorescent marker lines of *A. thaliana* as well as a review of the published literature of various plant species. The molecular and genetic mechanisms of the CP's ontogeny and MGU association are still unknown. The developmental analysis of different pollen mutants demonstrates that GC cytoplasmic extension ontogeny and MGU association are not controlled by *DUO1*, *DAZ1*, *DUO3*, *DAZ1 DAZ2*, *DUO2*, *FBL17*, *CDKA;1*, and *GUM* in *A. thaliana*. Based on the available data from different pollen mutants, it seems interesting that the initiation of CP and the MGU association might be regulated by the same genes or pathways during microgametogenesis in angiosperms. To unfold this mystery, we need to generate a male gametophyte mutant without CP and then developmental analysis for the existence of a MGU. If a MGU is still established in the absence of a CP, then both are independently regulated, otherwise not. Therefore, gametophytic screening of mutagenized marker lines may be a promising way forward to identify the structural and regulatory components that determine MGU assembly and CP development in future work.

## AUTHOR CONTRIBUTIONS

Experiments were conceived by AR and DT and executed by AR. The manuscript was written by AR and reviewed by JL and DT. The literature survey of MGU's existence was performed in BCP (AW, YL, ZL, MW) and TCP (IK, KJ, SW, MK).

## CONFLICT OF INTEREST STATEMENT

The authors declare no conflicts of interest.

## Supporting information


**Movie S1.** Male germ unit at early bicellular pollen. The spheroidal generative cell (green‐TET11‐GFP) has a cytoplasmic projection associated with the vegetative cell nuclear envelope (red‐LAT52:RanGAP‐tdTomato).

**Movie S2.** Male germ unit at mid‐bicellular pollen. The semi‐elongated generative cell (green‐ TET11‐GFP) has a cytoplasmic projection associated with the vegetative cell nuclear envelope (red‐LAT52:RanGAP‐tdTomato).

**Movie S3.** Male germ unit at late bicellular pollen. The fully elongated generative cell (green‐ TET11‐GFP) has a single long coiled cytoplasmic projection, which is linked in its distal region with the vegetative cell nuclear envelope (red‐LAT52:RanGAP‐tdTomato) or vegetative cell nucleus (red‐DUO3:H2B‐tdTomato).

**Movie S4.** Male germ unit at late bicellular pollen. The fully elongated generative cell (green‐ TET11‐GFP) has a single long coiled cytoplasmic projection, which is linked in its distal region with the vegetative cell nuclear envelope (red‐LAT52:RanGAP‐tdTomato) or vegetative cell nucleus (red‐DUO3:H2B‐tdTomato).

**Movie S5.** Male germ unit in tricellular pollen. The axially elongated pair of sperm cells (green‐TET11‐GFP) are connected with the vegetative cell nuclear envelope (red‐LAT52:RanGAP‐tdTomato), via a long cytoplasmic projection from one sperm cell (SC), while the other sperm cell has a small protuberance. The vegetative cell nuclear envelope has an irregular folded morphology.

**Movie S6.** Male germ unit in tricellular pollen. The axially elongated pair of sperm cells (green‐TET11‐GFP) are connected with the vegetative cell nuclear envelope (red‐LAT52:RanGAP‐tdTomato), via a long cytoplasmic projection from one sperm cell (SC), while the other sperm cell has a small protuberance. The vegetative cell nuclear envelope has an irregular folded morphology.

**Movie S7.** Male germ unit in tricellular pollen. A physical association of sperm cell (green‐TET11‐GFP) with the vegetative cell nucleus (red‐DUO3:H2B‐tdTomato), via a long cytoplasmic projection from one sperm cell (SC), while the other sperm cell has a small protuberance.

**Table S1.** Transgenic pollen markers used in this study. Markers 1 to 3 are male germline‐specific, marker 4 (LAT52:RanGAP‐tdTomato) is vegetative cell‐specific, while marker 5 (DUO3:H2B‐tdTomato) marks both the vegetative cell and male germline cells. These fluorescent markers were generated through Gateway recombinant technology.
**Table S2.** Transgenic markers and their expression in wild type and mutant pollen. These fluorescent markers i.e. TET11‐GFP, HTR10:GFP‐TUA6, DUO1:TET11‐tdTomato and LAT52:RanGAP‐tdTomato, expressed in wild‐type, male germ unit mutant (gum) and other bicellular pollen mutants (except duo1–2 & duo1–4). The duo1–2 and duo1–4 have no expression of HTR10:GFP‐TUA6 but DUO1:TET11‐tdTomato marks the plasma membrane of both alleles. The LAT52:RanGAP‐tdTomato and DUO3:H2B‐tdTomato have expression both in wild and mutant pollen. The method used to introduce markers into wild‐type and mutant lines is indicated as either crossed (C) or transformed (T).
**Table S3.** Mutant alleles used in this study. Detail of male germ unit mutants (gum) and other bicellular pollen mutants (cdka;1, fbl17, duo1–2, duo1–4, daz1–1 daz2–1, duo2 and duo3) their accession, locus, mutagenesis, and reference.
**Table S4.** Comparative fluorescence microscopic counting data of the germ unit malformed mutant (gum1–2−/−). The physical association between germline and vegetative nuclear membrane at different bud stages demonstrates that one SC of the pair has a physical association with the vegetative nuclear membrane (100%) at different bud stages (−4 to −1), irrespective of the apparently detached sperm cells from the vegetative nucleus in gum1–2−/− TET11‐GFP × LAT52:RanGAP‐tdTomato A4.
**Table S5.** Comparative counting data of the male germ unit association in gum1–1−/−. The physical association between germline and vegetative nuclear membrane at different bud stages demonstrates that one SC of the pair has physical contact with the vegetative nuclear membrane (100%) at different bud stages (−4 to −1), irrespective that the two sperm cells are positioned far from the vegetative nucleus in gum1–1−/− TET11‐GFP × LAT52:RanGAP‐tdTomato D4.
**Table S6.** List of various primers used for cloning of CDS, Promoter and Reporter genes through Multisite Gateway® recombination. In PCR1 the attB1 and attB2 sequences were added to CDS (attB1 (F)‐ 5'AC AAA GCA GGC TCG/attB2 (R)‐3'A CAA GAA AGC TGG GTA), while in PCR2 the Adapter attB1 and attB2 (Adapter Primer attB1 (F)‐5'GGGGACAAGTTTGT3’/Adapter Primer attB2 (R)‐ 3'ACAAAGTGGTCCCC5’) were added. (http://tools.invetrogen.com/content/sfs/manuals/gatewayman.pdf.).
**Table S7.** List shows details of different constructs and transformed plasmids used in this study by following Multisite Gateway® recombination cloning technology (http://tools.invetrogen.com/content/sfs/manuals/gatewayman.pdf.).

**Figure S1.** Close existence of the generative cell and the vegetative cell nucleus in wild‐type Arabidopsis pollen. Panels a‐e show DIC images of bicellular pollen from early (a) to late (e) bud stages prior to PM‐I of generative cells. The generative cell is round in profile and closely positioned with the vegetative cell nucleus even at early stages (a‐b). Axial elongation of the generative cell body occurs parallel to the vegetative nucleus (c‐e) and organelles that mark the cytoplasmic projection of the generative cell are seen wrapping around the vegetative cell nucleus (b‐e). Images are arranged from early to late bicellular pollen stages (Left to Right). Six independent wild‐type individuals were analyzed showing the same developmental pattern. n = 100 Spores per stage. Scale bar = 5 μm.
**Figure S2**. Cytoplasmic projection of the generative cell at different bicellular bud stages. The upper row represents DIC and the lower GFP (TET11‐GFP‐tags male germline plasma membrane). The round generative cell has a fine thread‐like cytoplasmic projection, which extends toward the vegetative nucleus (h), or the body of the round generative cell is closely positioned with the vegetative nucleus (a/f) at an early stage. The cytoplasmic projection of elongated generative cell grows parallel to the vegetative nucleus and appears to be associated with the vegetative nucleus (i‐j). Images are arranged from early to late bicellular pollen stages (Left to Right). Five independent wild‐type individuals were analyzed showing the same developmental pattern. n = 90 Spores per stage. Scale bar = 5 μm.
**Figure S3.** The male germ unit at different bicellular pollen stages in *Arabidopsis thaliana* . The fluorescence micrographs of wt‐TET11‐GFP × LAT52:RanGAP‐tdTomato show cytoplasmic projection elongation and its association with the vegetative nuclear membrane at different bicellular bud stages. The upper row represents GFP (TET11‐GFP‐tags male germline plasma membrane) and lower RFP (RanGAP‐tdTomato‐tags vegetative cell nuclear membrane). The round generative cell is in physical contact with the vegetative nuclear membrane (a) at an early stage. The cytoplasmic projection of the generative cell is associated with the vegetative nuclear membrane at all developmental bicellular stages (c‐e). Images are arranged from early to late bicellular pollen stages (Left to Right). Five independent wild‐type individuals were analyzed showing the same developmental pattern. n = 80 Spores per stage. Scale bar = 5 μm.
**Figure S4.** Micrographs showing potential male germ unit at tricellular pollen stages. The upper row represents DIC and the lower GFP (GFP‐TUA6‐tags male germline). The cytoplasmic projection of one of the SC appears to be associated with the vegetative nucleus at early, mid, and late tricellular pollen stages (a‐j). The male germ unit association occurs at all different tricellular pollen stages (a‐j). Images are arranged from early to late tricellular pollen stages (Left to Right). Five independent wild‐type individuals were analyzed showing the same developmental pattern. n = 110 Spores per stage. Scale bar = 5 μm.
**Figure S5.** Potential male germ unit association at different tricellular pollen stages. The upper row represents DIC and lower GFP (TET11‐GFP‐tags male germline plasma membrane). The sperm cell with a long cytoplasmic projection appears to have physical association with the vegetative nucleus at different tricellular pollen stages i.e. “potential” male germ unit at early, mid, and late tricellular pollen stages. Images are arranged from early to late tricellular pollen stages (Left to Right). Five independent wild‐type individuals were analyzed showing the same developmental pattern. n = 110 Spores per stage. Scale bar = 5 μm.
**Figure S6.** Double fluorescent markers showing the male germ unit association. The upper row shows GFP (GFP‐TUA6‐tags male germline MT/cytoplasm), and the lower row represents RFP (RanGAP‐tdTomato‐tags the vegetative cell nuclear membrane). A sperm cell with a long cytoplasmic projection has a physical association with the vegetative nuclear envelope and forms the male germ unit at early, mid, and late tricellular pollen stages (a‐e). Images are arranged from early to late tricellular pollen stages (Left to Right). Five independent wild‐type individuals were analyzed showing the same developmental pattern. n = 100 Spores per stage. Scale bar = 5 μm.
**Figure S7.** Fluorescence micrographs of wt‐HTR10:GFP‐TUA6 × DUO3:H2B‐tdTomato at different developmental stages. The 1st & 3rd rows represent GFP (GFP‐TUA6‐tags male germline MT) and 2nd & 4th RFP (H2B‐tdTomato‐tags both generative cell and vegetative cell nuclei) images. The microscopic analysis indicates that the male germ unit is positioned in the centre of the pollen grain at different bud stages in wild type (i‐t). The microspore nucleus (f), vegetative nucleus (g‐h), generative cell (d‐e/k‐l) and male germ unit (g‐j) are in the periphery of pollen at early bud stages. The vegetative nucleus (g‐j/p–t) and male germ unit (m‐t) are situated in the centre of the pollen grain at different bicellular and most tricellular pollen stages in wild‐type *A. thaliana* . Images are arranged from early to mature bud stages (Left to Right). The RFP signal is not visible in the male germline due to a large vegetative nucleus (r‐t). Images are arranged from early to late pollen stages (Left to Right). Five independent wild‐type individuals were analyzed showing the same developmental pattern. n = 80 Spores per stage. Scale bar = 5 μm.
**Figure S8.** The male germ unit association in duo1–4+/− at different tricellular pollen stages. The 1st & 3rd rows represent RFP (TET11‐tdTomato‐tags male germline plasma membrane) and the 2nd & 4th show GFP (RanGAP‐GFP‐tags vegetative cell nuclear membrane). The upper two rows represent wild type (a‐j) and the lower two rows show duo1–4+/− (a*‐j*). The male germ unit is present at various tricellular developmental stages (a‐e). Similarly, the mutant generative cell of duo1–4 is also associated with the vegetative nuclear membrane at comparative bud stages and forms the male germ unit (a*‐e*). The bicellular pollen mutant lacks generative cell division at different tricellular pollen stages (Images are arranged from early to late pollen stages (Left to Right). Five mutant individuals were analyzed showing the MGU. n = 90 Spores per stage. Scale bar = 5 μm.
**Figure S9.** The presence of male germ unit association in duo1–2+/− developmentally. The upper row shows RFP (TET11‐tdTomato‐tags male germline plasma membrane) and lower GFP (RanGAP‐GFP‐tags vegetative cell nuclear membrane). The mutant generative cell of duo1–2 is closely associated with the vegetative nuclear membrane at different bud stages and forms the male germ unit (a‐e). The bicellular pollen mutant lacks generative cell division at different tricellular pollen stages (Left to Right represents early to late pollen stages). Five mutant individuals were analyzed showing the MGU. n = 90 Spores per stage. Scale bar = 5 μm.
**Figure S10.** The male germ unit association in duo3+/− at different bud stages. The upper row shows GFP (GFP‐TUA6‐tags male germline MT) and lower RFP (RanGAP‐tdTomato‐tags vegetative cell nuclear membrane) images. The mutant generative cell is closely associated with the vegetative nuclear membrane at different bud stages as the male germ unit (a‐e). The bicellular pollen mutant lacks generative cell division at different tricellular pollen stages (Pollen arranged from early to late pollen stages (Left to Right). Five mutant individuals were analyzed showing the MGU. n = 120 Spores per stage. Scale bar = 5 μm.
**Figure S11.** Mutant daz1–1−/−daz2–1+/− showing the male germ unit association developmentally. The upper two rows represent the wild type and the lower rows show daz1–1−/− daz2–1+/. The 1st & 3rd rows represent GFP (GFP‐TUA6‐tags male germline MT/cytoplasm) and the 2nd & 4th RFP (RanGAP‐tdTomato‐tags vegetative cell nuclear membrane). One of the sperm cells is physically linked with the vegetative nuclear envelope through its cytoplasmic projection at various tricellular developmental stages (a‐e). Similarly, the mutant generative cell is associated with the vegetative nuclear membrane at comparative bud stages and forms the male germ unit (a*‐e*). The bicellular pollen mutant lacks generative cell division at different tricellular pollen stages (from early to late pollen stages‐Left to Right). Five mutant individuals were analyzed showing the MGU. n = 130 Spores per stage. Scale bar = 5 μm.
**Figure S12.** Male germ unit in duo2+/− at different tricellular pollen stages. The upper row shows GFP (GFP‐TUA6‐tags male germline MT) and lower RFP (RanGAP‐tdTomato‐tags vegetative cell nuclear membrane). The mutant generative cell lacks division and has the male germ unit association through cytoplasmic projection at different bud stages (a‐e). Images are arranged from early to late pollen stages (Left to Right). Five mutant individuals were analyzed showing the MGU. n = 120 Spores per stage. Scale bar = 5 μm.
**Figure S13.** Fluorescence micrographs of gum1–2−/−TET11‐GFP × DUO3:H2B‐tdTomato at different developmental stages. The upper row represents GFP (TET11‐GFP‐tags germline plasma membrane) and lower RFP (H2B‐tdTomato‐tags both generative cell and vegetative cell nuclei) images. One of the sperm cells appears to have physical contact with the vegetative nucleus and forms the male germ unit (MGU). The two sperm cells appear to be positioned far from the vegetative nucleus but appear to have physical contact with the vegetative nucleus in gum1–2 (b/g; e/j). The vegetative nucleus is near the pollen wall instead of the centre (h‐j). Images are arranged from early to mature stages (Left to Right). The RFP signal is not visible or weak in the male germline compared to that of the vegetative nucleus, where the RFP expression can be seen developmentally due to its large size or in the plane (g/i/j). Five mutant individuals were analyzed showing the MGU. n = 110 Spores per stage. Scale bar = 5 μm.
**Figure S14.** Fluorescence micrographs of gum1–1−/−TET11‐GFP × DUO3:H2B‐tdTomato at different developmental stages. The upper row represents GFP (TET11‐GFP‐tags male germline plasma membrane) and lower RFP (H2B‐tdTomato‐tags both generative cell and vegetative cell nuclei) images. One of the sperm cells appears to have physical contact with the vegetative nucleus and forms the male germ unit (MGU). The two sperm cells are positioned toward the pollen wall in the periphery, but one SC appears to have physical contact with the vegetative nucleus in gum1–1 (a‐e). The vegetative nucleus has an RFP signal and the male germline lacks RFP expression which may be due to the large size of the vegetative nucleus or in the plane (f‐j). Images are arranged from early to mature tricellular pollen stages (Left to Right). Five mutant individuals were analyzed showing the MGU. n = 110 Spores per stage. Scale bar = 5 μm.
**Figure S15.** Light and fluorescence micrographs of cdka;1+/− showing the male germ unit association developmentally. The upper row shows DIC, middle GFP (GFP‐TUA6‐tags male germline MT/cytoplasm) and lower RFP (RanGAP‐tdTomato‐tags vegetative cell nuclear membrane). The mutant generative cell of cdka;1+/− lacks division and is physically linked with the vegetative nuclear membrane through cytoplasmic projection at different bud stages and forms the male germ unit (f‐j). Images are arranged from early to late pollen stages (Left to Right). Five mutant individuals were analyzed showing the MGU. n = 120 Spores per stage. Scale bar = 5 μm.

## Data Availability

The data are available from the corresponding author on request.
