# Supplementary material for: The male germ unit association is independently regulated of GUM in Arabidopsis thaliana
Source: Plant Direct. 2024 Jul 29;8(7):e624. doi: 10.1002/pld3.624 (PMC11286290; doi:10.1002/pld3.624)
Supplement: Supplementary file 9 — Figure S1. Close existence of the generative cell and the vegetative cell nucleus in wild‐type Arabidopsis pollen. Panels a‐e show DIC images of bicellular pollen from early (a) to late (e) bud stages prior to PM‐I of generative cells. The generative cell is round in profile and closely positioned with the vegetative cell nucleus even at early stages (a‐b). Axial elongation of the generative cell body occurs parallel to the vegetative nucleus (c‐e) and organelles that mark the cytoplasmic projection of the generative cell are seen wrapping around the vegetative cell nucleus (b‐e). Images are arranged from early to late bicellular pollen stages (Left to Right). Six independent wild‐type individuals were analyzed showing the same developmental pattern. n = 100 Spores per stage. Scale bar = 5 μm. Figure S2. Cytoplasmic projection of the generative cell at different bicellular bud stages. The upper row represents DIC and the lower GFP (TET11‐GFP‐tags male germline plasma membrane). The round generative cell has a fine thread‐like cytoplasmic projection, which extends toward the vegetative nucleus (h), or the body of the round generative cell is closely positioned with the vegetative nucleus (a/f) at an early stage. The cytoplasmic projection of elongated generative cell grows parallel to the vegetative nucleus and appears to be associated with the vegetative nucleus (i‐j). Images are arranged from early to late bicellular pollen stages (Left to Right). Five independent wild‐type individuals were analyzed showing the same developmental pattern. n = 90 Spores per stage. Scale bar = 5 μm. Figure S3. The male germ unit at different bicellular pollen stages in Arabidopsis thaliana . The fluorescence micrographs of wt‐TET11‐GFP × LAT52:RanGAP‐tdTomato show cytoplasmic projection elongation and its association with the vegetative nuclear membrane at different bicellular bud stages. The upper row represents GFP (TET11‐GFP‐tags male germline plasma membrane) and lower RFP (RanGA [file PLD3-8-e624-s004.docx]

**Table S1. Transgenic pollen markers used in this study.** Markers 1 to 3 are male germline-specific, marker 4 (LAT52:RanGAP-tdTomato) is vegetative cell-specific, while marker 5 (DUO3:H2B-tdTomato) marks both the vegetative cell and male germline cells. These fluorescent markers were generated through Gateway recombinant technology.

| Marker | | Marker expression in germline | | | Marker expression in vegetative cell | | Reference |
| --- | --- | --- | --- | --- | --- | --- | --- |
|  |  | Plasma membrane | Cytoplasm/MT | Nucleus | Nucleus | Nuclear membrane |  |
| 1 | TET11-GFP | √ | **×** | **×** | **×** | **×** | Boavida *et al.,* 2013 |
| 2 | DUO1:TET11-tdTomato | √ | **×** | **×** | **×** | **×** | This study |
| 3 | HTR10:GFP-TUA6 | **×** | √ | **×** | **×** | **×** | Ueda *et al.,* 1999;  Khatab, 2012 |
| 4 | LAT52:RanGAP-tdTomato | **×** | **×** | **×** | **×** | √ | Rose & Meier, 2001; this study |
| 5 | DUO3:H2B-tdTomato | **×** | **×** | √ | √ | **×** | Brownfield *et al.*, 2009b |

**Table S2. Transgenic markers and their expression in wild type and mutant pollen.** These fluorescent markers i.e. TET11-GFP, HTR10:GFP-TUA6, DUO1:TET11-tdTomato and LAT52:RanGAP-tdTomato, expressed in wild-type, male germ unit mutant (*gum*) and other bicellular pollen mutants (except *duo1-2* & *duo1-4*). The *duo1-2* and *duo1-4* have no expression of HTR10:GFP-TUA6 but DUO1:TET11-tdTomato marks the plasma membrane of both alleles. The LAT52:RanGAP-tdTomato and DUO3:H2B-tdTomato have expression both in wild and mutant pollen. The method used to introduce markers into wild-type and mutant lines is indicated as either crossed (C) or transformed (T).

| Genotype | Germline markers | VC markers | Method (C, crossed; T, transformed) |
| --- | --- | --- | --- |
| WT | TET11-GFP, HTR10:GFP-TUA6 |  | C |
|  | DUO1:TET11-tdTomato  DUO3:H2B-tdTomato | DUO3:H2B-tdTomato, LAT52:RanGAP-tdTomato | T |
| *duo2^+/-^* | HTR10:GFP-TUA6 |  | C |
|  |  | LAT52:RanGAP-tdTomato | T |
| *duo3^+/-^* | HTR10:GFP-TUA6 |  | C |
|  |  | LAT52:RanGAP-tdTomato | T |
| *daz1-1^-/-^daz2-1^+/-^* | HTR10:GFP-TUA6 |  | C |
|  |  | LAT52:RanGAP-tdTomato | T |
| *duo1-2^+/-^, duo1-4^+/-^* | DUO1:TET11-tdTomato | LAT52:RanGAP-tdTomato | T |
| *fbl17^+/-^* | HTR10: GFP-TUA6 |  | C |
|  |  | LAT52:RanGAP-tdTomato | T |
| *cdka;1^+/-^* | HTR10: GFP-TUA6 |  | C |
|  |  | LAT52:RanGAP-tdTomato | T |
| *gum1-1 ^+/-^*  *gum 1-2 ^+/-^* | TET11-GFP, HTR10:GFP-TUA6 |  | C |
|  | DUO1:TET11-tdTomato,  DUO3:H2B-tdTomato | DUO3:H2B-tdTomato  LAT52:RanGAP-tdTomato | T |

**Table S3. Mutant alleles used in this study**. Detail of male germ unit mutants (gum) and other bicellular pollen mutants (*cdka;1*, *fbl17*, *duo1-2*, *duo1-4*, *daz1-1 daz2-1*, *duo2* and *duo3*) their accession, locus, mutagenesis, and reference.

| Mutant allele | Accession | Locus | Mutagen and lesion | Reference |
| --- | --- | --- | --- | --- |
| *gum1-1,*  *gum1-2* | No-0 |  | EMS, Chrom-IV between markers M506-SSLP (21.9 cM) and nga8 (26.6 cM) | Lalanne and Twell, 2002 |
| *cdka;1* | Col-0 | AT3G48750 | T-DNA insertion at +1816 bp (SALK_106809) | Iwakawa et al., 2006; Nowack et al., 2006 |
| *fbl17* | Col-0 | AT3G54650 | T-DNA insertion at +1278 bp (GABI_170E02) | Kim et al., 2008 |
| *duo1-2* | C24 | AT3G60460 | Gamma-rays, 14 bp insertion at +672 bp | Rotman et al., 2005 |
| *duo1-4* | Col-0 | AT3G60460 | EMS, C>T at +545 bp | Borg et al., 2014 |
| *daz1-1 daz2-1* | Col-0 | AT2G17180 | daz1-1 (SALK_058012),  daz1-2 (SALK_151422) | Scholl et al., 2000 |
| *duo2* | No-0 |  |  | Durbarry et al., 2005 |
| *duo3* | No-0 | AT1G64570 |  | Durbarry et al., 2005; Brownfield *et al.*, 2009b |

**Table S4. Comparative fluorescence microscopic counting data of the germ unit malformed mutant (*gum1-2^-/-^*).** The physical association between germline and vegetative nuclear membrane at different bud stages demonstrates that one SC of the pair has a physical association with the vegetative nuclear membrane (100%) at different bud stages (-4 to -1), irrespective of the apparently detached sperm cells from the vegetative nucleus in *gum1-2^-/-^ TET11-GFP×LAT52:RanGAP-tdTomato* A4.

| Bud Stages | Intact association b/w SC and VN | Dissociated linkage b/w SC and VN |
| --- | --- | --- |
| Bud-1 | 140 | 0 |
| Bud-2 | 124 | 0 |
| Bud-3 | 135 | 0 |
| Bud-4 | 118 | 0 |

**Table S5. Comparative counting data of the male germ unit association in *gum1-1^-/-^*.** The physical association between germline and vegetative nuclear membrane at different bud stages demonstrates that one SC of the pair has physical contact with the vegetative nuclear membrane (100%) at different bud stages (-4 to -1), irrespective that the two sperm cells are positioned far from the vegetative nucleus in *gum1-1^-/-^ TET11-GFP × LAT52:RanGAP-tdTomato* D4.

| Bud Stages | Intact association b/w SC and VN | Dissociated linkage b/w SC and VN |
| --- | --- | --- |
| Bud-1 | 118 | 0 |
| Bud-2 | 136 | 0 |
| Bud-3 | 120 | 0 |
| Bud-4 | 110 | 0 |

**Table S6.** List of various primers used for cloning of CDS, Promoter and Reporter genes through Multisite Gateway® recombination. In PCR1 the attB1 and attB2 sequences were added to CDS (attB1 (F)- 5'AC AAA AAA GCA GGC TCG/ attB2 (R)-3'A CAA GAA AGC TGG GTA), while in PCR2 the Adapter attB1 and attB2 (Adapter Primer attB1 (F)-5'GGGGACAAGTTTGT3'/ Adapter Primer attB2 (R)- 3'ACAAAGTGGTCCCC5') were added. (http://tools.invetrogen.com/content/sfs/manuals/gatewayman.pdf.).

| Name | Part used | Forward Primer | Reverse Primer |
| --- | --- | --- | --- |
| TET11 no stop attB1 & 2 | CDS | 5' AC AAA AAA GCA GGC TCGATGTTTCGAGTTAGCAATTTC 3' | 3' A CAA GAA AGC TGG GTAGACAGAATCACTTTTCCTAGCC 5' |
| TET11 attB4 & 1 | Promoter | 5' TG TAT AGA AAA GTT GCATTTCATTTTTCCATATCAAATGTAC 3' | 3' T TTT GTA CAA ACT TGCTTTTGGAAATTTGCTTTCTCC 5' |
| RanGAP1-WPP-attB1 & 2 | CDS | 5'AAAAAGCAGGCTCTATGGATCATTCAGCGAAAACC 3' | 3'ACAAGAAAGCTGGGTCCTCAACCTCGGATTCTTCCTG 5' |
| H2B attB1 & 2 | CDS | 5'AAAAAGCAGGCTCAATGGCGAAGGCAGATAAGAAACC3' | 3'AGAAAGCTGGGTCCCAGCTCCAGCAGAACTCGTAAAC5' |
| Lat52 attB4 & 1 | Promoter | 5'TGTATAGAAAAGTTGTTGAGGAATGATCGATTCTGG3' | 3'TTTTGTACAAACTTGGAAATTTTTTTTTTGGTGTGTG5' |
| DUO3 attB4 & 1 | Promoter | 5'TGTATAGAAAAGTTGCGATGATGGGGTTTCTAATCCAG3' | 3'TTTTGTACAAACTTGGACAACAATTGCAATTCTCAAAC5' |
| mGFP attB2 & 3 | Reporter | 5'TCTTGTACAAAGTGGAAATGAGTAAAGGAGAAGAACTT3' | 3'TGTATAATAAAGTTGTTATTTGTATAGTTCATCCATGCC5' |
| mRFP attB2 & 3 | Reporter | 5'TCTTGTACAAAGTGGCGATGGCCTCCTCCGAGGACG3' | 3'TGTATAATAAAGTTGTTAGGCGCCGGTGGAGTGG5' |

**Table S7.** List shows details of different constructs and transformed plasmids used in this study by following Multisite Gateway® recombination cloning technology (http://tools.invetrogen.com/content/sfs/manuals/gatewayman.pdf.).

| Plasmid name + Construct/ Cloned Fragment | Construct or cloned fragment |
| --- | --- |
| pK7m34GW ProDUO3:H2B-tdTomato | ProDUO3:H2B-tdTomato |
| pK7m34GW ProDUO3:H2B-GFP | ProDUO3:H2B-GFP |
| pK7m34GW ProLAT52:RanGAP-tdTomato | ProLAT52:RanGAP-tdTomato |
| pK7m34GW ProLAT52:RanGAP-GFP | ProLAT52:RanGAP-GFP |
| pDONR221 RanGAP-WPP | RanGAP-WPP |
| pH7m34GW ProDUO1:TET11-tdTomato | ProDUO1:TET11-tdTomato |
| pDONR 221 TET11 CDS | TET11 CDS |
| pDONRP2RP3 GFP | GFP |
| pDONRP2RP3 m RFP | m RFP |

**
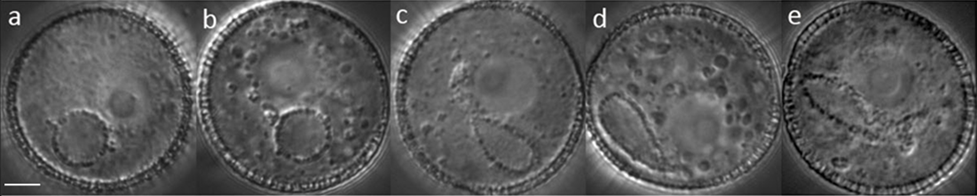
**

**Figure S1. Close existence of the generative cell and the vegetative cell nucleus in wild-type Arabidopsis pollen.** Panels a-e show DIC images of bicellular pollen from early (a) to late (e) bud stages prior to PM-I of generative cells. The generative cell is round in profile and closely positioned with the vegetative cell nucleus even at early stages (a-b). Axial elongation of the generative cell body occurs parallel to the vegetative nucleus (c-e) and organelles that mark the cytoplasmic projection of the generative cell are seen wrapping around the vegetative cell nucleus (b-e). Images are arranged from early to late bicellular pollen stages (Left to Right). Six independent wild-type individuals were analysed showing the same developmental pattern. n=100 Spores per stage. Scale bar = 5 µm

**
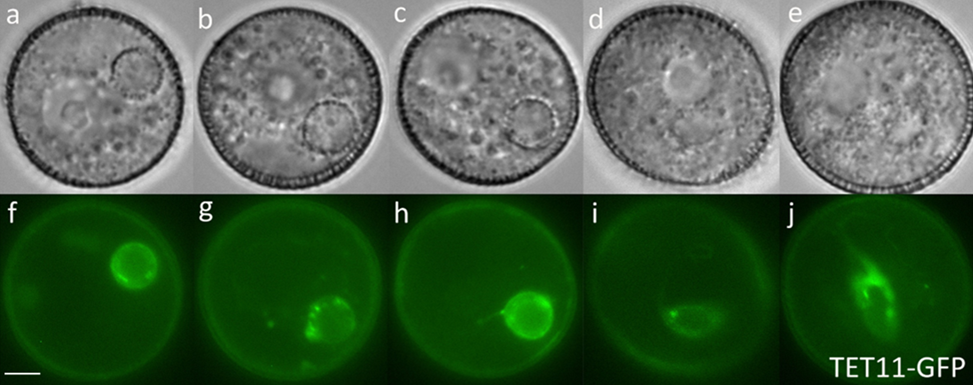
**

**Figure S2. Cytoplasmic projection of the generative cell at different bicellular bud stages**. The upper row represents DIC and the lower GFP (TET11-GFP-tags male germline plasma membrane). The round generative cell has a fine thread-like cytoplasmic projection, which extends towards the vegetative nucleus (h), or the body of the round generative cell is closely positioned with the vegetative nucleus (a/f) at an early stage. The cytoplasmic projection of elongated generative cell grows parallel to the vegetative nucleus and appears to be associated with the vegetative nucleus (i-j). Images are arranged from early to late bicellular pollen stages (Left to Right). Five independent wild-type individuals were analysed showing the same developmental pattern. n=90 Spores per stage. Scale bar = 5µm

**
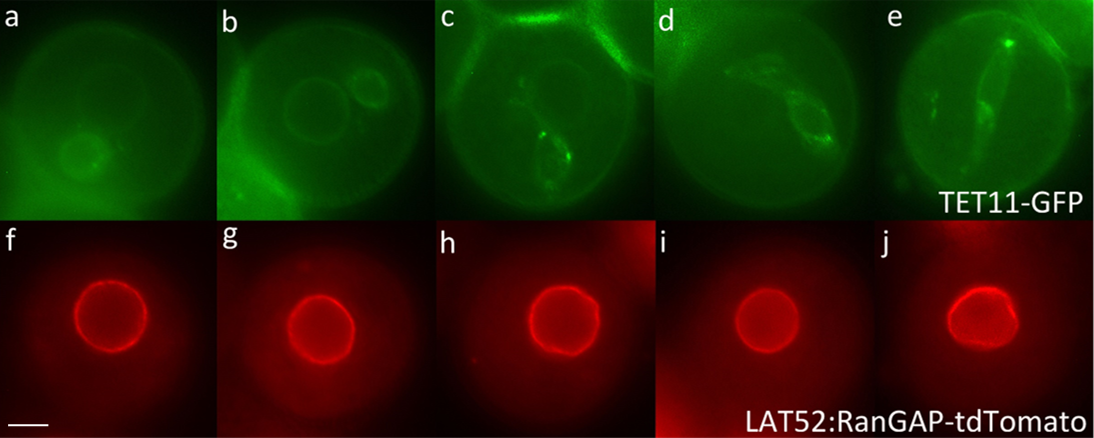
**

**Figure S3. The male germ unit at different bicellular pollen stages in *Arabidopsis thaliana*.** The fluorescence micrographs of wt-TET11-GFP×LAT52:RanGAP-tdTomato show cytoplasmic projection elongation and its association with the vegetative nuclear membrane at different bicellular bud stages. The upper row represents GFP (TET11-GFP-tags male germline plasma membrane) and lower RFP (RanGAP-tdTomato-tags vegetative cell nuclear membrane). The round generative cell is in physical contact with the vegetative nuclear membrane (a) at an early stage. The cytoplasmic projection of the generative cell is associated with the vegetative nuclear membrane at all developmental bicellular stages (c-e). Images are arranged from early to late bicellular pollen stages (Left to Right). Five independent wild-type individuals were analysed showing the same developmental pattern. n=80 Spores per stage. Scale bar = 5µm

**
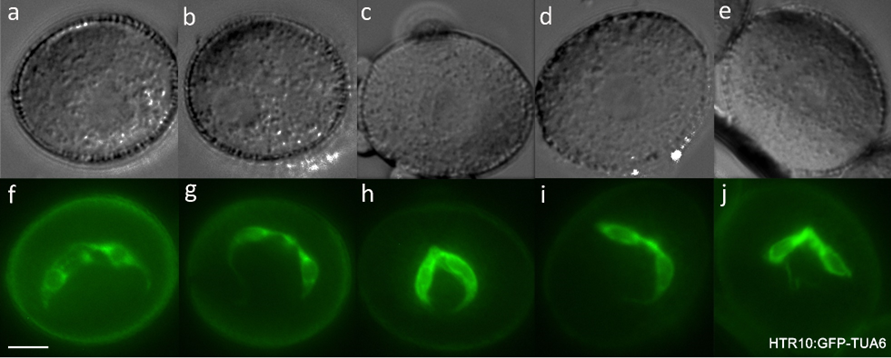
**

**Figure S4. Micrographs showing potential male germ unit at tricellular pollen stages.** The upper row represents DIC and the lower GFP (GFP-TUA6-tags male germline). The cytoplasmic projection of one of the SC appears to be associated with the vegetative nucleus at early, mid, and late tricellular pollen stages (a-j). The male germ unit association occurs at all different tricellular pollen stages (a-j). Images are arranged from early to late tricellular pollen stages (Left to Right). Five independent wild-type individuals were analysed showing the same developmental pattern. n=110 Spores per stage. Scale bar = 5µm

**
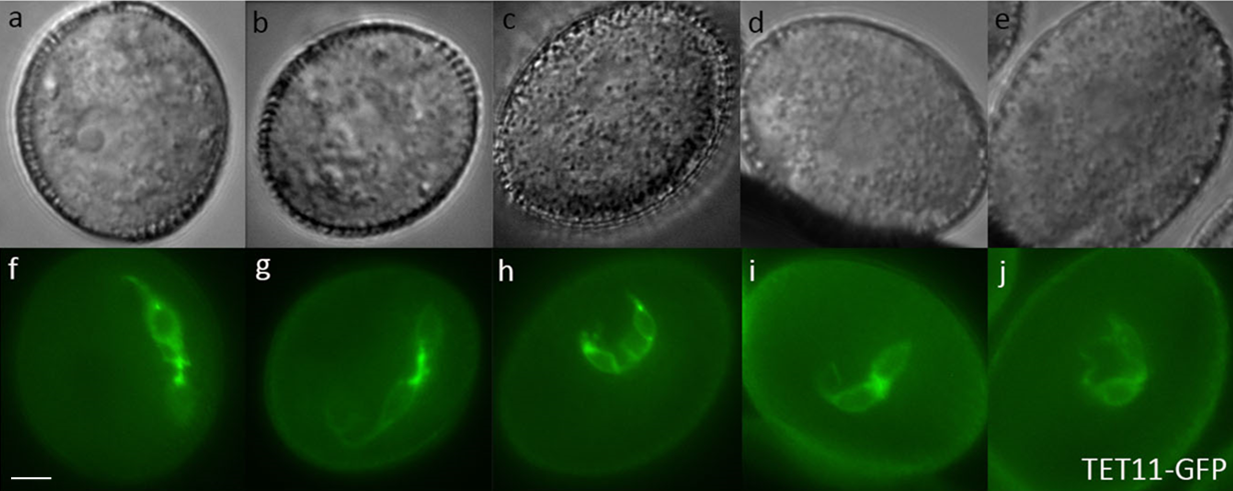
**

**Figure S5. Potential male germ unit association at different tricellular pollen stages.** The upper row represents DIC and lower GFP (TET11-GFP-tags male germline plasma membrane). The sperm cell with a long cytoplasmic projection appears to have physical association with the vegetative nucleus at different tricellular pollen stages i.e. "potential" male germ unit at early, mid, and late tricellular pollen stages. Images are arranged from early to late tricellular pollen stages (Left to Right). Five independent wild-type individuals were analysed showing the same developmental pattern. n=110 Spores per stage. Scale bar = 5µm

**
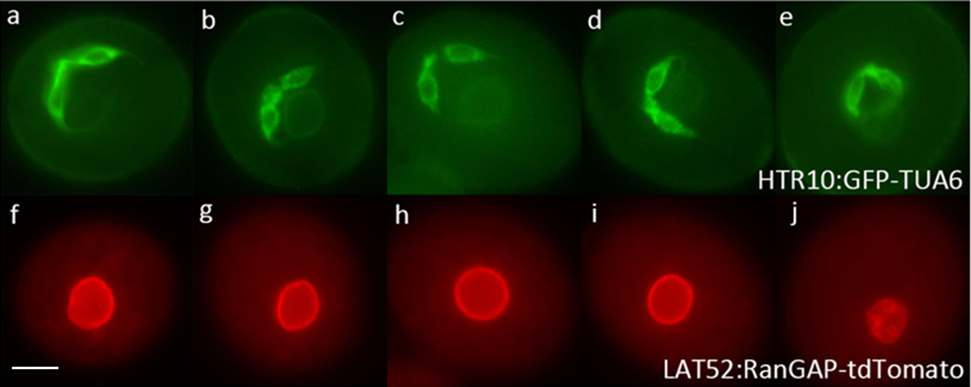
**

**Figure S6. Double fluorescent markers showing the male germ unit association.** The upper row shows GFP (GFP-TUA6-tags male germline MT/cytoplasm), and the lower row represents RFP (RanGAP-tdTomato-tags the vegetative cell nuclear membrane). A sperm cell with a long cytoplasmic projection has a physical association with the vegetative nuclear envelope and forms the male germ unit at early, mid, and late tricellular pollen stages (a-e). Images are arranged from early to late tricellular pollen stages (Left to Right). Five independent wild-type individuals were analysed showing the same developmental pattern. n=100 Spores per stage. Scale bar = 5µm

**
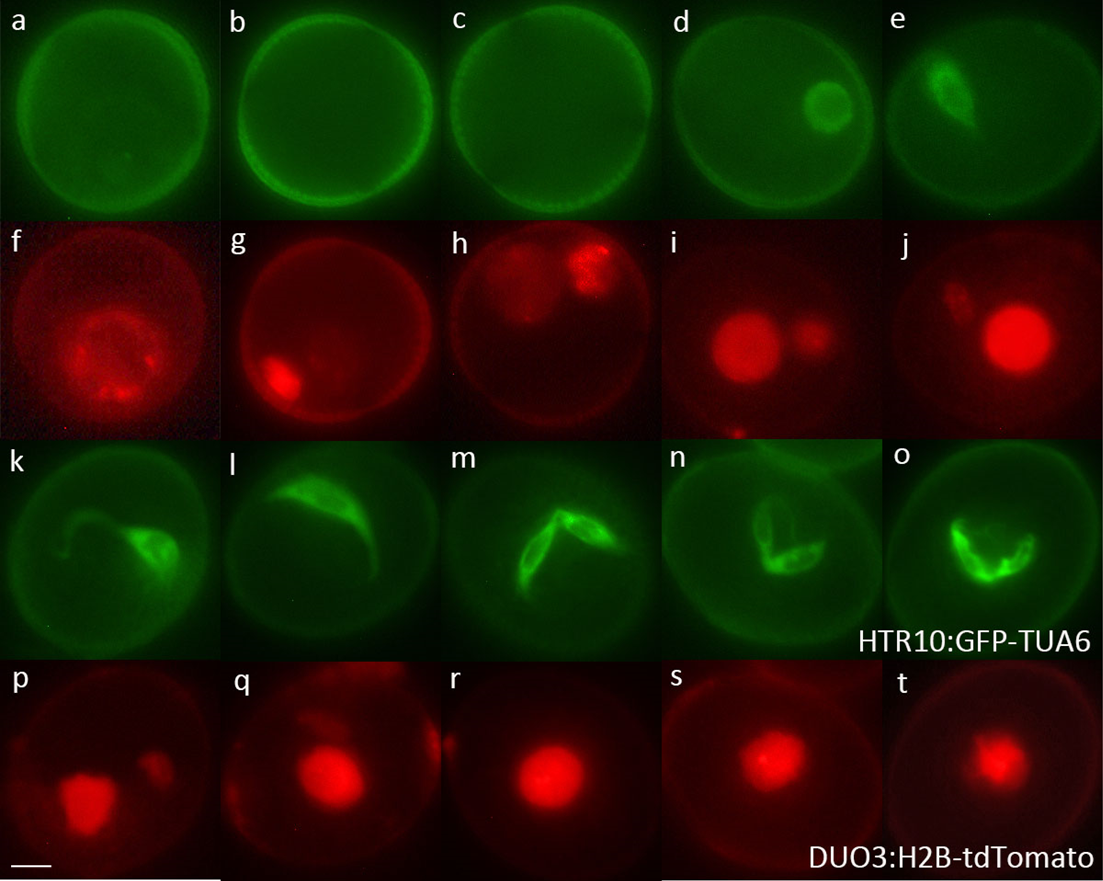
**

**Figure S7. Fluorescence micrographs of wt-HTR10:GFP-TUA6×DUO3:H2B-tdTomato at different developmental stages.** The 1^st^ & 3^rd^ rows represent GFP (GFP-TUA6-tags male germline MT) and 2^nd^ & 4^th^ RFP (H2B-tdTomato-tags both generative cell and vegetative cell nuclei) images. The microscopic analysis indicates that the male germ unit is positioned in the centre of the pollen grain at different bud stages in wild type (i-t). The microspore nucleus (f), vegetative nucleus (g-h), generative cell (d-e/k-l) and male germ unit (g-j) are in the periphery of pollen at early bud stages. The vegetative nucleus (g-j/p-t) and male germ unit (m-t) are situated in the centre of the pollen grain at different bicellular and most tricellular pollen stages in wild-type *Arabidopsis thaliana*. Images are arranged from early to mature bud stages (Left to Right). The RFP signal is not visible in the male germline due to a large vegetative nucleus (r-t). Images are arranged from early to late pollen stages (Left to Right). Five independent wild-type individuals were analysed showing the same developmental pattern. n=80 Spores per stage. Scale bar = 5µm

**
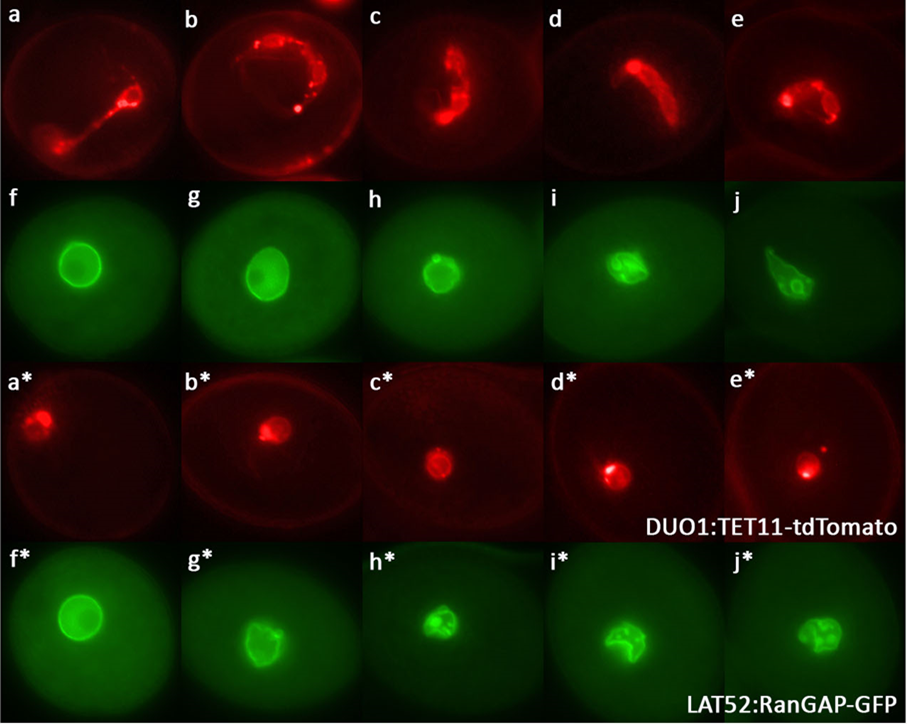
**

**Figure S8. The male germ unit association in *duo1-4^+/-^* at different tricellular pollen stages.** The 1^st^ & 3^rd^ rows represent RFP (TET11-tdTomato-tags male germline plasma membrane) and the 2^nd^ & 4^th^ show GFP (RanGAP-GFP-tags vegetative cell nuclear membrane). The upper two rows represent wild type (a-j) and the lower two rows show *duo1-4^+/-^* (a*-j*). The male germ unit is present at various tricellular developmental stages (a-e). Similarly, the mutant generative cell of *duo1-4* is also associated with the vegetative nuclear membrane at comparative bud stages and forms the male germ unit (a*-e*). The bicellular pollen mutant lacks generative cell division at different tricellular pollen stages (Images are arranged from early to late pollen stages (Left to Right). Five mutant individuals were analysed showing the MGU. n=90 Spores per stage. Scale bar = 5µm


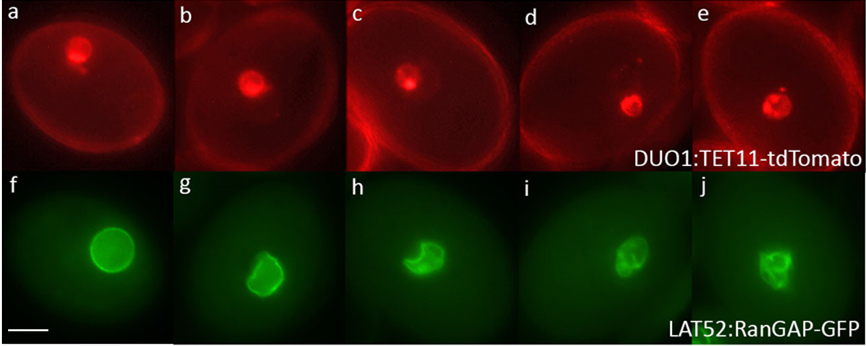


**Figure S9. The presence of male germ unit association in *duo1-2^+/-^* developmentally**. The upper row shows RFP (TET11-tdTomato-tags male germline plasma membrane) and lower GFP (RanGAP-GFP-tags vegetative cell nuclear membrane). The mutant generative cell of *duo1-2* is closely associated with the vegetative nuclear membrane at different bud stages and forms the male germ unit (a-e). The bicellular pollen mutant lacks generative cell division at different tricellular pollen stages (Left to Right represents early to late pollen stages). Five mutant individuals were analysed showing the MGU. n=90 Spores per stage. Scale bar = 5µm

**
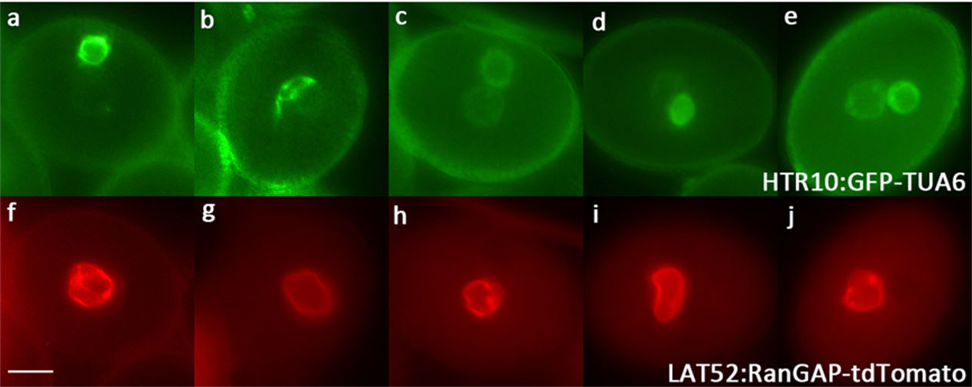
**

**Figure S10. The male germ unit association in *duo3^+/-^* at different bud stages.** The upper row shows GFP (GFP-TUA6-tags male germline MT) and lower RFP (RanGAP-tdTomato-tags vegetative cell nuclear membrane) images. The mutant generative cell is closely associated with the vegetative nuclear membrane at different bud stages as the male germ unit (a-e). The bicellular pollen mutant lacks generative cell division at different tricellular pollen stages (Pollen arranged from early to late pollen stages (Left to Right). Five mutant individuals were analysed showing the MGU. n=120 Spores per stage. Scale bar = 5µm

**
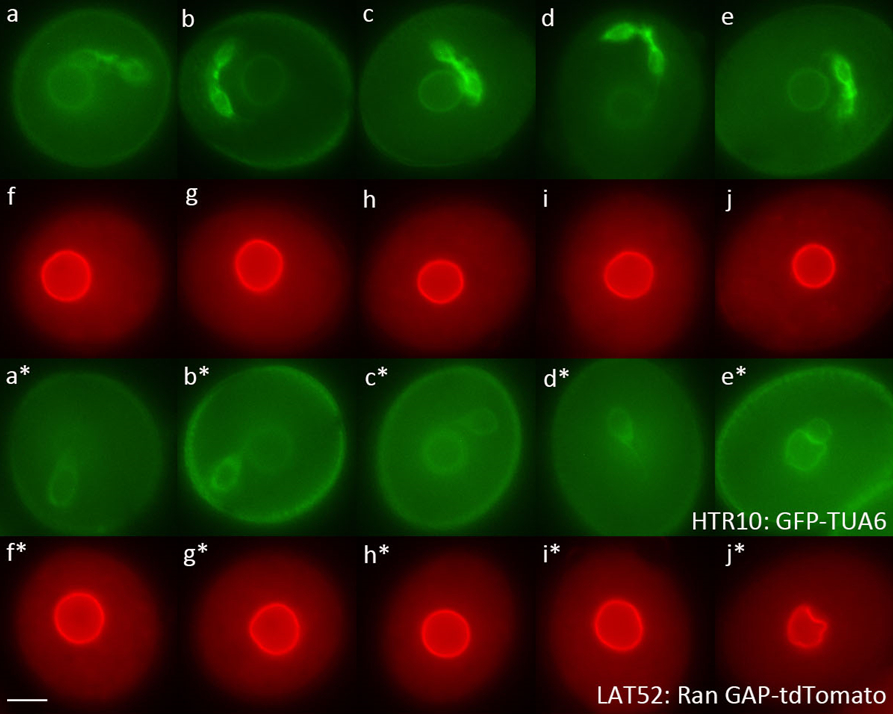
**

**Figure S11. Mutant *daz1-1^-/-^daz2-1^+/-^* showing the male germ unit association developmentally.** The upper two rows represent the wild type, and the lower rows show *daz1-1^-/-^ daz2-1^+/^.* The 1^st^ & 3^rd^ rows represent GFP (GFP-TUA6-tags male germline MT/cytoplasm) and the 2^nd^ & 4^th^ RFP (RanGAP-tdTomato-tags vegetative cell nuclear membrane). One of the sperm cells is physically linked with the vegetative nuclear envelope through its cytoplasmic projection at various tricellular developmental stages (a-e). Similarly, the mutant generative cell is associated with the vegetative nuclear membrane at comparative bud stages and forms the male germ unit (a*-e*). The bicellular pollen mutant lacks generative cell division at different tricellular pollen stages (from early to late pollen stages-Left to Right). Five mutant individuals were analysed showing the MGU. n=130 Spores per stage. Scale bar = 5µm

**
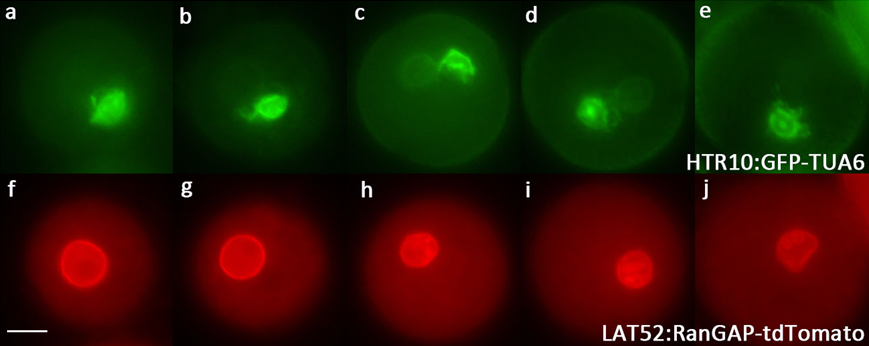
**

**Figure S12. Male germ unit in *duo2^+/-^* at different tricellular pollen stages.** The upper row shows GFP (GFP-TUA6-tags male germline MT) and lower RFP (RanGAP-tdTomato-tags vegetative cell nuclear membrane). The mutant generative cell lacks division and has the male germ unit association through cytoplasmic projection at different bud stages (a-e). Images are arranged from early to late pollen stages (Left to Right). Five mutant individuals were analysed showing the MGU. n=120 Spores per stage. Scale bar = 5µm

**
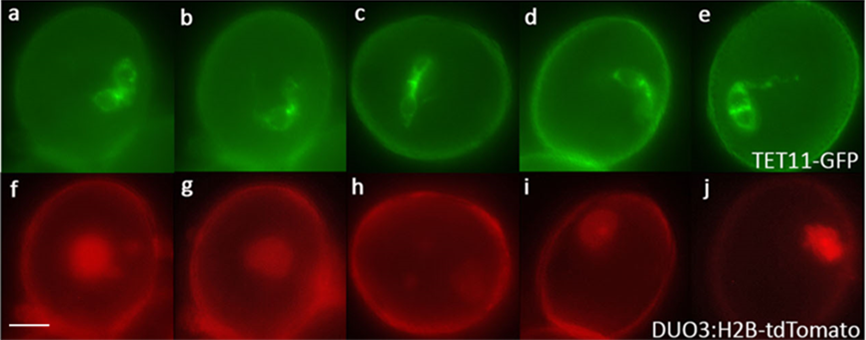
**

**Figure S13. Fluorescence micrographs of *gum1-2^-/-^TET11-GFP×DUO3:H2B-tdTomato* at different developmental stages.** The upper row represents GFP (TET11-GFP-tags germline plasma membrane) and lower RFP (H2B-tdTomato-tags both generative cell and vegetative cell nuclei) images. One of the sperm cells appears to have physical contact with the vegetative nucleus and forms the male germ unit (MGU). The two sperm cells appear to be positioned far from the vegetative nucleus but appear to have physical contact with the vegetative nucleus in *gum1-2* (b/g; e/j). The vegetative nucleus is near the pollen wall instead of the centre (h-j). Images are arranged from early to mature stages (Left to Right). The RFP signal is not visible or weak in the male germline compared to that of the vegetative nucleus, where the RFP expression can be seen developmentally due to its large size or in the plane (g/i/j). Five mutant individuals were analysed showing the MGU. n=110 Spores per stage. Scale bar = 5 µm

**
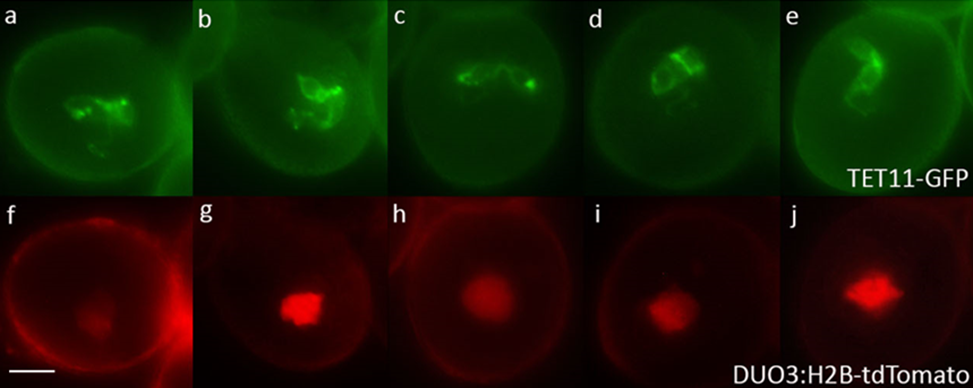
**

**Figure S14. Fluorescence micrographs of *gum1-1^-/-^TET11-GFP×DUO3:H2B-tdTomato* at different developmental stages**. The upper row represents GFP (TET11-GFP-tags male germline plasma membrane) and lower RFP (H2B-tdTomato-tags both generative cell and vegetative cell nuclei) images. One of the sperm cells appears to have physical contact with the vegetative nucleus and forms the male germ unit (MGU). The two sperm cells are positioned towards the pollen wall in the periphery, but one SC appears to have physical contact with the vegetative nucleus in *gum1-1* (a-e). The vegetative nucleus has an RFP signal, and the male germline lacks RFP expression, possibly due to the large size of the vegetative nucleus or in the plane (f-j). Images are arranged from early to mature tricellular pollen stages (Left to Right). Five mutant individuals were analysed showing the MGU. n=110 Spores per stage. Scale bar = 5µm

**
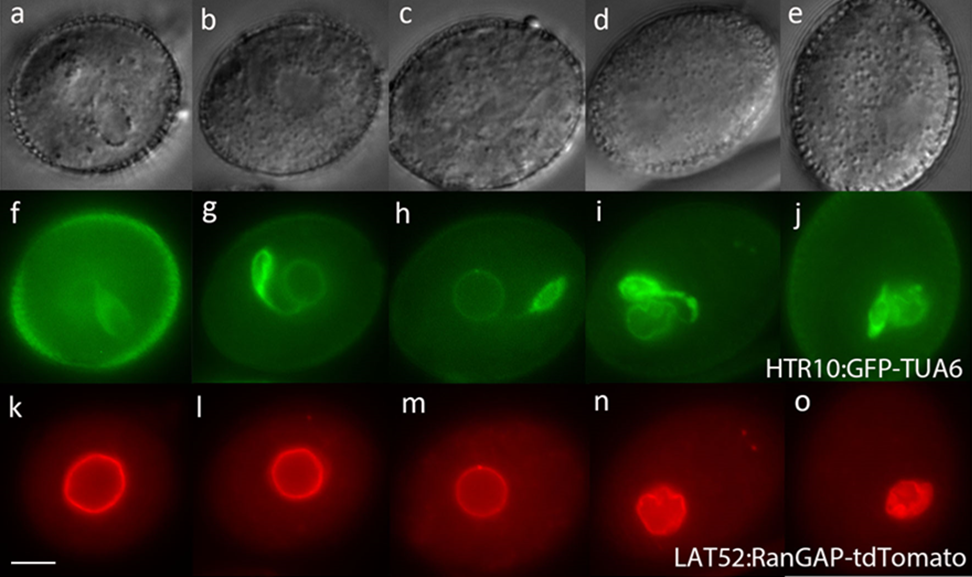
**

**Figure S15. Light and fluorescence micrographs of *cdka;1^+/-^* show the development of the male germ unit association.** The upper row shows DIC, middle GFP (GFP-TUA6-tags male germline MT/cytoplasm) and lower RFP (RanGAP-tdTomato-tags vegetative cell nuclear membrane). The mutant generative cell of *cdka;1^+/-^* lacks division and is physically linked with the vegetative nuclear membrane through cytoplasmic projection at different bud stages and forms the male germ unit (f-j). Images are arranged from early to late pollen stages (Left to Right). Five mutant individuals were analysed showing the MGU. n=120 Spores per stage. Scale bar = 5µm

**References**

Boavida, L.C., Qin, P., Broz, M., Becker, J.D., & McCormick, S. (2013) Arabidopsis tetraspanins are confined to discrete expression domains and cell types in reproductive tissues and form homo- and heterodimers when expressed in yeast. *Plant Physiology*, 163: 696-712.

Borg, M., Rutley, N., Kagale, S., Hamamura, Y., Gherghinoiu, M., Kumar, S., Sari, U., Esparza-Franco, M.A., Sakamoto, W., Rozwadowski, K., Higashiyama, T., & Twell, D. (2014). An EAR-Dependent Regulatory Module Promotes Male Germ Cell Division and Sperm Fertility in Arabidopsis. *The Plant Cell,* 26(5), pp. 2098-2113.

Brownfield, L., Hafidh, S., Durbarry, A., Khatab, H., Sidorova, A., Doerner, P., & Twell, D. (2009b). Arabidopsis DUO POLLEN3 is a key regulator of male germline development and embryogenesis. *The Plant Cell*, 21: 1940-1956.

Durbarry, A., Vizir, I. & Twell, D. (2005). Male germ line development in Arabidopsis. duo pollen mutants reveal gametophytic regulators of generative cell cycle progression. *Plant Physiology,* 137(1), pp. 297-307.

Iwakawa, H., Shinmyo, A., & Sekine, M. (2006). Arabidopsis cdka;1, a cdc2 homologue, controls proliferation of generative cells in male gametogenesis. *The Plant Journal*, 45(5), 819-831. <https://doi.org/10.1111/j.1365-313x.2005.02643.x>

Khatab, H.A. (2012) Molecular and genetic mechanisms regulating sperm cell development in Arabidopsis thaliana. PhD Thesis, University of Leicester.

Kim, H.J., Oh, S.A., Brownfield, L., Hong, S.H., Ryu, H., Hwang, I., Twell, D., & Nam, H.G. (2008). Control of plant germline proliferation by SCFFBL17 degradation of cell cycle inhibitors. *Nature,* 455(7216), pp. 1134-1137.

Lalanne, E., & Twell, D. (2002). Genetic control of male germ unit organization in Arabidopsis. *Plant Physiology,* 129(2), pp. 865-875.

Nowack, M.K., Grini, P.E., Jakoby, M.J., Lafos, M., Koncz, C., & Schnittger, A. (2006). A positive signal from the fertilization of the egg cell sets off endosperm proliferation in angiosperm embryogenesis. *Nat. Genet*. 38, 63–67.

Rose, A., & Meier, I. (2001) A domain unique to plant RanGAP is responsible for its targeting to the plant nuclear rim. Proceedings of the National Academy of Sciences of the United States of America, 98: 15377-15382.

Rotman, N., Durbarry, A., Wardle, A., Yang, W.C., Chaboud, A., Faure, J., Berger, F. & Twell, D. (2005) 'A novel class of MYB factors controls sperm-cell formation in plants', *Current Biology,* 15(3), pp. 244-248.

Scholl, R., May, S.T., & Ware, D. (2000). Seed and molecular resources for Arabidopsis. *Plant physiology, 124 4*, 1477-80.

Ueda, K., Matsuyama, T., & Hashimoto, T. (1999) Visualization of microtubules in living cells of transgenic Arabidopsis thaliana. *Protoplasma*, 206: 201-206.
