# Supplementary material for: The male germ unit association is independently regulated of GUM in Arabidopsis thaliana
Source: Plant Direct. 2024 Jul 29;8(7):e624. doi: 10.1002/pld3.624 (PMC11286290; doi:10.1002/pld3.624)
Supplement: Supplementary file 8 — Table S1. Transgenic pollen markers used in this study. Markers 1 to 3 are male germline‐specific, marker 4 (LAT52:RanGAP‐tdTomato) is vegetative cell‐specific, while marker 5 (DUO3:H2B‐tdTomato) marks both the vegetative cell and male germline cells. These fluorescent markers were generated through Gateway recombinant technology. Table S2. Transgenic markers and their expression in wild type and mutant pollen. These fluorescent markers i.e. TET11‐GFP, HTR10:GFP‐TUA6, DUO1:TET11‐tdTomato and LAT52:RanGAP‐tdTomato, expressed in wild‐type, male germ unit mutant (gum) and other bicellular pollen mutants (except duo1–2 & duo1–4). The duo1–2 and duo1–4 have no expression of HTR10:GFP‐TUA6 but DUO1:TET11‐tdTomato marks the plasma membrane of both alleles. The LAT52:RanGAP‐tdTomato and DUO3:H2B‐tdTomato have expression both in wild and mutant pollen. The method used to introduce markers into wild‐type and mutant lines is indicated as either crossed (C) or transformed (T). Table S3. Mutant alleles used in this study. Detail of male germ unit mutants (gum) and other bicellular pollen mutants (cdka;1, fbl17, duo1–2, duo1–4, daz1–1 daz2–1, duo2 and duo3) their accession, locus, mutagenesis, and reference. Table S4. Comparative fluorescence microscopic counting data of the germ unit malformed mutant (gum1–2−/−). The physical association between germline and vegetative nuclear membrane at different bud stages demonstrates that one SC of the pair has a physical association with the vegetative nuclear membrane (100%) at different bud stages (−4 to −1), irrespective of the apparently detached sperm cells from the vegetative nucleus in gum1–2−/− TET11‐GFP × LAT52:RanGAP‐tdTomato A4. Table S5. Comparative counting data of the male germ unit association in gum1–1−/−. The physical association between germline and vegetative nuclear membrane at different bud stages demonstrates that one SC of the pair has physical contact with the vegetative nuclear membrane (100%) at different bud stag [file PLD3-8-e624-s009.pdf]

Plant Direct
Open Access

The Male Germ Unit association is independently regulated of GUM in *Arabidopsis thaliana*

Abdur Rauf[1,2,3,4*], Anbang Wang[1], Yujia Li[1], Zhihao Lian[1], Shouxing Wei[1], Kashmala Jabbar[2], Muhammad Wisal[2], Ikramullah Khan[2], Muhammad Khalid[5], Jingyang Li [1,4*]

Decision Letter Round 1:

June 10, 2024

Dr. Abdur Rauf
Haikou Experimental Station, Chinese Academy of Tropical Agricultural Sciences
Haikou, N/A
China

RE: Male Germ Unit association is independently regulated of GUM in Arabidopsis thaliana

Dear Dr. Rauf:

Thank you for submitting to Plant Direct. All required reviews have been returned and we have now finished our evaluation of your manuscript. In light of the reviewers' and editor's comments, further revisions are needed before the paper can be accepted for publication in Plant Direct.

Please view the editors' and reviewers' comments below and use their suggestions as a guide while you work on your revision.

When uploading the revised version of this article, please be sure to include the following:

-A word document that contains your response to the reviewers. You should respond to each reviewer comment and note the changes made to the manuscript. If you do not agree with a reviewer's comment and choose not to make a suggested revision, please explain why. Please try to provide as complete an answer as possible to each reviewer's criticisms.
-A tracked changes document with each change highlighted
- A clean version of the latest version of the manuscript

The manuscript provides interesting data and overall is technically sound. However, both reviewers mentioned the need for better description and justification of some experiments.

More important, the conclusions should be strongly supported by the data presented. While Plant Direct does not normally ask for extra experimental work, it is important that the conclusions are based on experimental evidence and that the limitations of the work are stated and any speculation is presented as a hypothesis.

To upload your revision, please click the link below.
https://plantdirect.msubmit.net/cgi-bin/main.plex?el=A3Lr5hS2A3okG3I6A9ftd2DoIMhiZSCPdPueKUDxAZ

In order to provide as timely a service as possible, we ask that your revision is resubmitted within three months after receipt of this request. If an extension is needed, please send a request, along with a brief explanation, to the editorial office at plantdirect@wiley.com .

Please note that, in addition to publishing reviewer comments, the author's responses to review comments will also be published alongside the final version of the paper. If you would not like the author's responses to be published, please contact the editorial office at plantdirect@wiley.com .

Thank you very much for giving us an opportunity to review your work. I look forward to receiving the next version.

Sincerely,

Gustavo MacIntosh

Gustavo MacIntosh

Editor, Plant Direct

--- Editor comments:
The manuscript provides interesting data and overall is technically sound. However, both reviewers mentioned the need for better description and justification of some experiments.
More important, the conclusions should be strongly supported by the data presented. While Plant Direct does not normally ask for extra experimental work, it is important that the conclusions are based on experimental evidence and that the limitations of the work are stated

and any speculation is presented as a hypothesis.

-- Reviewer comments:
Reviewer #1:

This manuscript describes the cytoplasmic connections between the components of the male germ unit and their importance in the assembly of a functional reproductive unit. It is an important manuscript in the field because it may help us better understand the physical interactions between the vegetative nucleus and the sperm or generative cells in the pollen tube.

The main finding of this manuscript is that the cytoplasmic connections between the generative cell (in bicellular pollen) or a sperm cell (in tricellular pollen) with the vegetative nucleus are lost when some mutations are introduced. This is an important finding, although it raises some questions that the authors leave unanswered. For example, the authors mentioned that one of the mutant proteins might act as a regulator of the cytoskeleton, and I think it would be helpful to analyze the cytoskeleton of mutant generative or sperm cells to see if the increased distance between them and the vegetative nucleus is due to changes in cytoskeletal organization.

A question about the abstract. At the end, the authors mentioned some specific proteins that supposedly act as transcription factors (DUO1-DAZ1 and DUO3). These proteins have never been mentioned before. So, I think more information is needed to make the reader aware of their importance in this context.

The introduction sufficiently describes the background context of the manuscript and provides essential information. I have only one comment. At the end of the introduction, I think more information is needed to present the experimental approach used by the authors to analyze the physical connections between the vegetative nucleus and the sperm cells.
In addition, in the Materials and Methods, the authors described the analysis of genes such as DUO, DAZ, GUM, and so on. But these genes and proteins were not mentioned in the Introduction. So, more information is needed to introduce these proteins to the readers.
The use of GFP marker lines is only briefly mentioned, but no technical information is provided on how they were obtained. This is in striking contrast to the great (sometimes excessive) detail provided for microscopic analysis.

The authors should explain why they are using some specific markers (HTR10:GFP-TUA6×DUO3:H2B-tdTomato; HTR10:GFP134 TUA6×LAT52:RanGAP-tdTomato) and what they represent. Otherwise, it is difficult for the reader to follow the text.

On line 152, the authors introduced another mutant (mud) without explaining what it is and how it might be useful in this study. Please keep in mind that not all readers are familiar with these terms. A figure showing the localization and putative role of all the proteins analyzed by the authors might be useful for the reader.

Based on their observations, the authors concluded on line 170 that GUM may regulate the cytoskeleton or cellular network. How did they conclude that GUM is a regulator of the cytoskeleton? And what do the authors mean for the cellular network?

The drawing in Figure 6 is clear, but one piece of information is missing. What do the black dots represent? It is not specified in the figure legend. The model should be more informative if other proteins studied in this manuscript are added. This will help readers interpret the results.

Reviewer #2:

The manuscript by Rauf and Li "Male Germ Unit association is independently regulated of GUM in Arabidopsis thaliana" addresses the development of the MGU and its dynamic organization in
developing pollen of wild-type and in several independent mutants. However, our knowledge about the establishment of the CP and MGU association remains limited.
Overall, the main weakness is the lack of quantitative data, as the consequence this work comes as highly descriptive. For example, when author says "Length of the cytoplasmic projection increases with GC body morphogenesis and an elongated cytoplasmic projection has been observed at the late bicellular stage (Fig. 1b, Fig. S2-S3)", or "One of the SCs has a long cytoplasmic projection and the other has a small (80%) or two cytoplasmic projections of equal length (20%) in A. thaliana." Is it possible to quantify this?
Overall, I think the manuscript would benefit from a more detailed description of the images to make it more accessible to non-experts. For example, it would be useful to show the different development stages and/or to include a scheme to facilitate the interpretation of them.
Finally, in my opinion the conclusions section does not reflect the main findings.

Decision Letter Round 2:

July 6, 2024
Dr. Abdur Rauf

Plant Direct
Open Access

Haikou Experimental Station, Chinese Academy of Tropical Agricultural Sciences
Haikou, N/A
China

MSID: 2024-01482TWR1
MS TITLE: Male Germ Unit association is independently regulated of GUM in Arabidopsis thaliana

Dear Dr. Abdur Rauf:

I am pleased to inform you that your manuscript "Male Germ Unit association is independently regulated of GUM in Arabidopsis thaliana" has been accepted for publication in Plant Direct.

Your article will appear online in the next available issue of Plant Direct. To ensure your article gets published as quickly as possible, please pay attention to the steps detailed below. We have found that most of the delays happen at this stage, especially at the payment stage, so please respond as quickly as possible when prompted.

License Agreement: Once your article has been accepted it will move to Production and undergo admin and file checking - you may receive an email with any queries we have at this stage. When all required items are received by the publisher and queries resolved, the corresponding author will receive an email from Wiley's Author Services system which will ask them to log on at https://authorservices.wiley.com/bauthor and will present them with the appropriate license for completion. Your article cannot be published until both the signed license agreement and payment of the article fee have been received.

Payment of the Open Access Article Publication Fee: All articles published in Plant Direct are fully open access: immediately and freely available to read, download and share. Plant Direct charges a publication fee to cover the publication costs. The corresponding author for this manuscript should have already received a quote from the payments team (cs-openaccess@wiley.com) with the estimated article publication fee; please email cs-openaccess@wiley.com if this has not been received. The corresponding author should log on to the Wiley Author Services site, where the publication fee can be paid by credit card or an invoice. Pro Forma can also be requested. Payment of the publication charge must be received before the article will be published online.

Proofs: You will have the opportunity to look over your paper once more when you receive the author proofs for your article. The proofs will be with you in approximately two weeks. Please

note that, in addition to publishing reviewer comments, the author's responses to review comments will also be published alongside the final version of the paper. If you would not like the author's responses to be published, please contact the editorial office at plantdirect@wiley.com .

Promotion of your article: You can help your research get the attention it deserves! Check out Wiley's free Promotion Guide for best-practice recommendations for promoting your work at www.wileyauthors.com/eeo/guide. And learn more about Wiley Editing Services which offers professional video, design, and writing services to create shareable video abstracts, infographics, conference posters, lay summaries, and research news stories for your research at www.wileyauthors.com/eeo/promotion.

Thank you again for your contribution to Plant Direct. If you have any questions, feel free to contact the editorial office at plantdirect@wiley.com .

Sincerely,

Gustavo MacIntosh

Gustavo MacIntosh

Editor, Plant Direct

Author's response to reviewer:

Reviewer #1 comments on the MS Plant Direct: 2024-01482TW and authors response

S.No	Reviewer comments	Authors Response
	**All updated changes are highlighted in Red in the MS suggested by the Reviewer please**	
1	One of the mutant proteins might act as a regulator of the cytoskeleton, and I think it would be helpful to analyze the cytoskeleton of mutant generative or sperm cells to see if the increased distance between them and the vegetative nucleus is due to changes in cytoskeletal organization	I like the idea, but it is far beyond this current paper, while our focus was to find the physical association between germline (GC/SC) and vegetative nucleus through cytoplasmic projection (gum) as well as the MGU association in wild-type Arabidopsis at bicellular pollen stages. In the future we need to create a male germ unit mutant, which has no physical contact between germline (GC/SC) and vegetative

		nucleus. This will provide the genetic regulation for this association and then the proposed mutant needs to be explored for the cytoskeleton network by the introduction of a fluorescent marker expressed with a vegetative cell-specific promoter to analyze the distorted network for its potential role to keep MGU components closed to each. In our current MS, we will include your ideas in the conclusion section for future research work. The analysis of vegetative cell cytoskeleton in *gum* is also an important idea.
**2**	A question about the abstract. At the end, the authors mentioned some specific proteins that supposedly act as transcription factors (DUO1-DAZ1 and DUO3). These proteins have never been mentioned before. So, I think more information is needed to make the reader aware of their importance in this context	Yes, that true that these transcription factors were not introduced in the MS, which I have updated now as per your advice.
**3**	At the end of the introduction, I think more information is needed to present the experimental approach used by the authors to analyze the physical connections between the vegetative nucleus and the sperm cells.	That is a very valid idea and has been added as suggested, please.
**4**	In addition, in the Materials and Methods, the authors described the analysis of genes such as DUO, DAZ, GUM, and so on. But these genes and proteins were not mentioned in the Introduction.	As per your suggestion, we added a summary of all these mutants to the introduction of the Revised MS (Highlighted in red).
**5**	The use of GFP marker lines is only briefly mentioned, but no technical information is provided on how they were obtained.	History and detailed information of these constructs is given in Tab. S1, with their source and expression in the plasma membrane, cytoplasm/MT, nucleus, and nuclear membrane

		of male gametophyte. Furthermore, markers transformed into different mutants were either generated at Twell Lab (HTR10:H2B-RFP, HTR10:TUA6-GFP, HTR10:H2B:GFP) or crossed into these mutants (wt TET11-GFP) or new markers were produced through Multisite Gateway® recombination (DUO1:TET11-tdTomato/GFP, LAT52:RanGAP-GFP/tdTomato, DUO3:H2B-tdTomato). Details of primers (Tab. S6) and Plasmid vectors (Tab. S7) are added in the Supplementary information as per your suggestion, please.
**6**	The authors should explain why they are using some specific markers (HTR10:GFP-TUA6×DUO3:H2B-tdTomato; HTR10:GFP134 TUA6×LAT52:RanGAP-tdTomato) and what they represent. Otherwise, it is difficult for the reader to follow the text.	Different fluorescent markers were introduced into the germline (GC/SC) and vegetative nucleus (VN) in male germ unit mutants (*gum1-1*, *gum1-2,* and *mud1*), bicellular pollen mutants (*duo1-2, duo1-4, daz1daz2, duo2, duo3, fbl17, cdka;1*) and wild type. This was performed to visualize the association of MGU through *in vivo* fluorescence microscopy. The strategy was to label male germline plasma membrane (TET11-GFP/DUO1:TET11-tdTomato), cytoplasm/MT (HTR10:GFP-TUA6) and vegetative nucleus (LAT52:H2B-mRFP/DUO3:H2B-tdTomato) or vegetative nuclear membrane (LAT52:RanGAP-tdTomato). Homozygous contrast double marker lines were generated in all these mutants, having these constructs in the given combination (i.e. HTR10:GFP-TUA6×DUO3:H2B-tdTomato, HTR10:GFP-TUA6×LAT52:RanGAP-tdTomato, TET11-GFP×DUO3:H2B-tdTomato and TET11-GFP×LAT52:RanGAP-tdTomato). Detailed information of these constructs is given in Tab. S1.
**7**	On line 152, the authors introduced another mutant (mud) without	Twell and Lalanne, 2002, analyzed two mutants i.e. *mud* and *gum*. In *mud*, the whole male germ

	explaining what it is and how it might be useful in this study. Please keep in mind that not all readers are familiar with these terms.	unit was displaced from the center to the periphery. We were expecting that the *mud* would have an intact MGU and with the introduction of fluorescent markers, an intact MGU was found. Therefore, that data is not presented in this MS as *mud* has no potential role in the integration of MGU. We added a summary of all these mutants to the introduction of Revised MS (Note: Developmental data of mud is presented at the end)
**8**	The authors concluded on line 170 that GUM may regulate the cytoskeleton or cellular network. How did they conclude that GUM is a regulator of the cytoskeleton? And what do the authors mean for the cellular network?	This was proposed that in the germ unit malformed (gum) mutant, the physical contact between the sperm cell (SC) and vegetative nucleus (VN) appears to be lost and therefore GUM might encode proteins to keep the SC and VN linked and/or close to each other (Lalanne and Twell 2002). In wildtype the MGU is normally positioned in the center (Fig. 1a), therefore it is presumed that GUM may regulate cytoskeleton or cellular network which keep the two components of the MGU close to each other but not organisation and assembly of the MGU, while in the absence of GUM, the SC and VN distance from each other.
**9**	The drawing in Figure 6 is clear, but one piece of information is missing. What do the black dots represent? It is not specified in the figure legend. The model should be more informative if other proteins studied in this manuscript are added. This will help readers interpret the results	We observed small lipid droplets (Black Dots), which have a high concentration on the surfaces of the plasma membrane (GC/SC). These are helpful markers in the DIC microscopy at different bicellular pollen stages. A description is added to the legend of Fig. 6 as suggested.

Serial Number_07-Additional Explanation

MGU association in *mud1*: In *mud1* the intact male germ unit is displaced from the center to the pollen periphery and located near the pollen wall (Lalanne and Twell, 2002) (Fig. 1). The homozygous germline marker individuals of *mud1* and wildtype (TET11-GFP×DUO3:H2B-tdTomato) have been analysed developmentally at different bud stages. The intact MGU is

positioned centrally (MGU Cen) in wildtype WT *Arabidopsis thaliana* based on *in vivo* developmental analysis at different tricellular and bicellular bud stages (Fig. 2). While in *mud1* the MGU has been displaced toward the periphery (MGU Per) and positioned near the wall of the pollen grain, and this shift of the MGU from the center towards periphery is maximum at different tricellular bud stages (Fig. 3). The analysis shows the dislocation of an intact male germ unit toward periphery can be seen in most of the pollen at the tricellular bud stages in *mud1*(Fig. 2). This shows that the loss of function of *MUD1* results in the displacement of the male germ unit from the center toward the periphery and positioned near the pollen wall. This might be due to the disruption of cellular networks that maintain the position of the vegetative nucleus and male germ unit in the center of the pollen grain, regulated by *MUD1*.

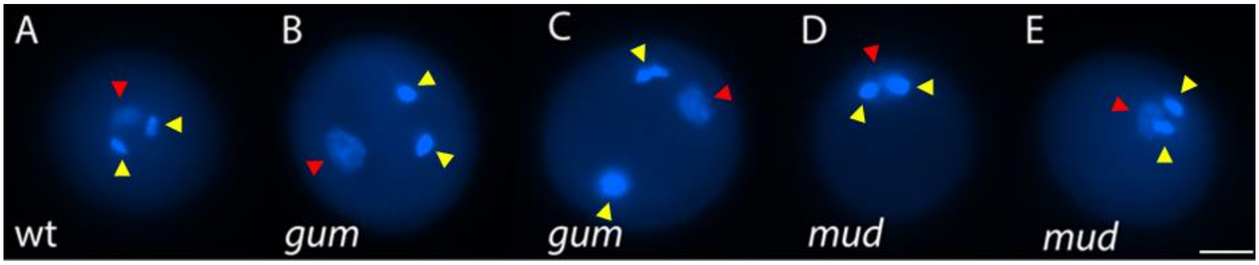

Figure 1. DAPI stained microscopic images of wild type (A), gum (B-C) and *mud* (D-E) showing the position of the MGU components. The two sperm nuclei are located in the centre close to the vegetative nucleus in wild type (A), while in *mud* both components are positioned in the periphery of pollen wall (D-E) but in *gum* the association between two sperm nuclei and vegetative nucleus appears to be lost (B-C). Arrowheads show position of male germline (yellow) or VC nuclei (red) inside the pollen grain. Scale bar =5µm (Lalanne and Twell 2002).

Note: (%MGU Cen= Percent of the MGU located centrally, % MGU Per= Percent of the MGU located peripheral)

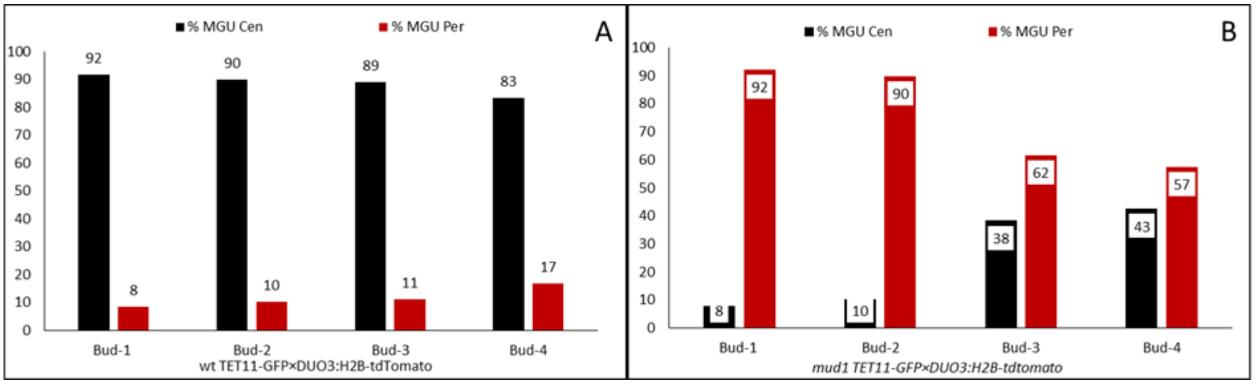

Figure 2. Comparative fluorescence microscopic counting data regarding position of the male germ unit in WT and *mud1* double marker lines (TET11-GFP$^{+/+}$×DUO3:H2B-tdTomato$^{+/+}$) at different bud stages (-4 to -1). The intact MGU is positioned centrally in majority of pollen grain at different tricellular and some bicellular bud stages i.e. mid and late bicellular (83-92%) in wild

type *Arabidopsis thaliana* (A). The centrally located male germ unit (WT) appears to be displaced toward periphery (MGU Per) and positioned near pollen wall and this shift of the MGU from centre towards periphery is maximum at different tricellular bud stages in *mud1* (B). Displacement of the MGU initiates at bicellular stages in some of the pollen and peripheral localisation of the intact male germ unit can be seen in majority of the pollen at tricellular bud stages in *mud1*(B). (WT n=112 (-4 bud stage), n=114 (-3 bud stage), n=109 (-2 bud stage), n=120 (-1 bud stage)/ *mud1* n=94 (-4 bud stage), n=107 (-3 bud stage), n=98 (-4 bud stage), n=96 (-1 bud stage).

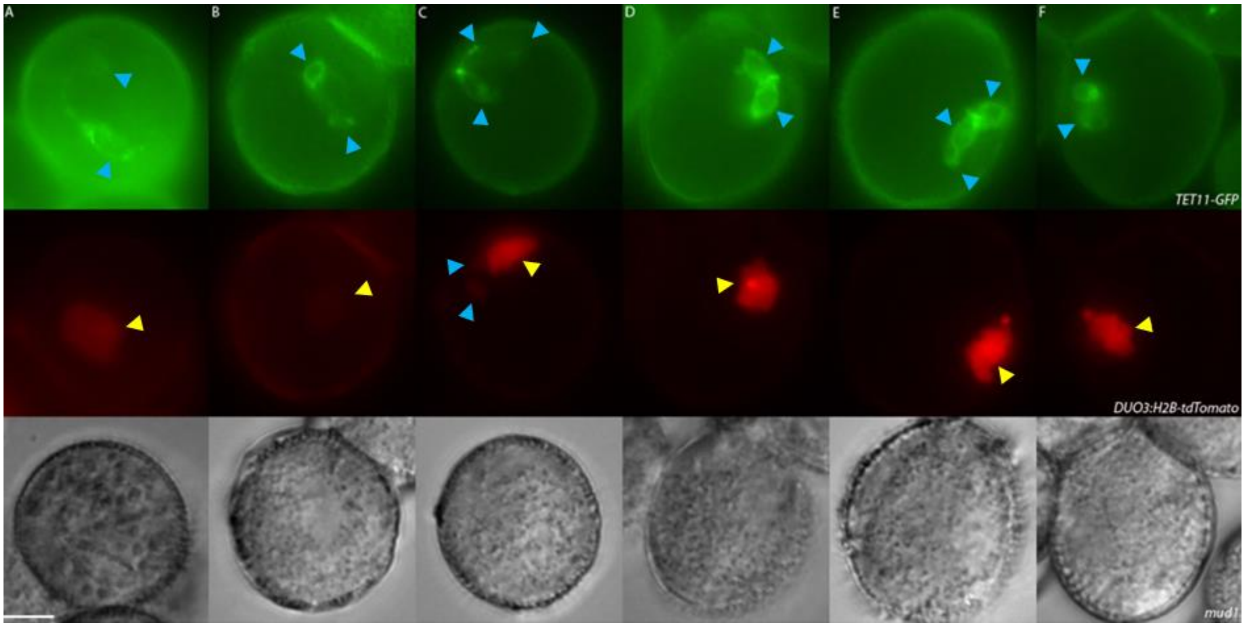

Figure 3. Light and fluorescence micrographs of *mud1*$^{-/-}$ *TET11-GFP*$^{+/+}$×*DUO3:H2B-tdTomato*$^{+/+}$ at different developmental stages. The upper row represents GFP (TET11-GFP-tags male germline plasma membrane), middle RFP (DUO3:H2B-tdTomato-tags both GC and VC nuclei) and lower DIC images. The intact MGU appears to be displaced from the centre toward periphery and positioned near the pollen wall in *mud1* (B-F). An association exists between the SC pair and VN in the displaced male germ unit (MGU). Mutant SCs bodies appear to lack elongated shape (D-F). Images are arranged from early to mature bud stages. Arrowheads show position of male germline (blue) or VC nuclei (yellow) inside the pollen grain. Scale bar =5µm

We also generate homozygous marker lines of *mud1* (*TET11-GFP×LAT52:RanGAP-tdTomato* / *DUO1:TET11-tdTomato×LAT52:RanGAP-GFP*) to exclude any artefact and our developmental analysis shows the same results (Fig. 4-5).

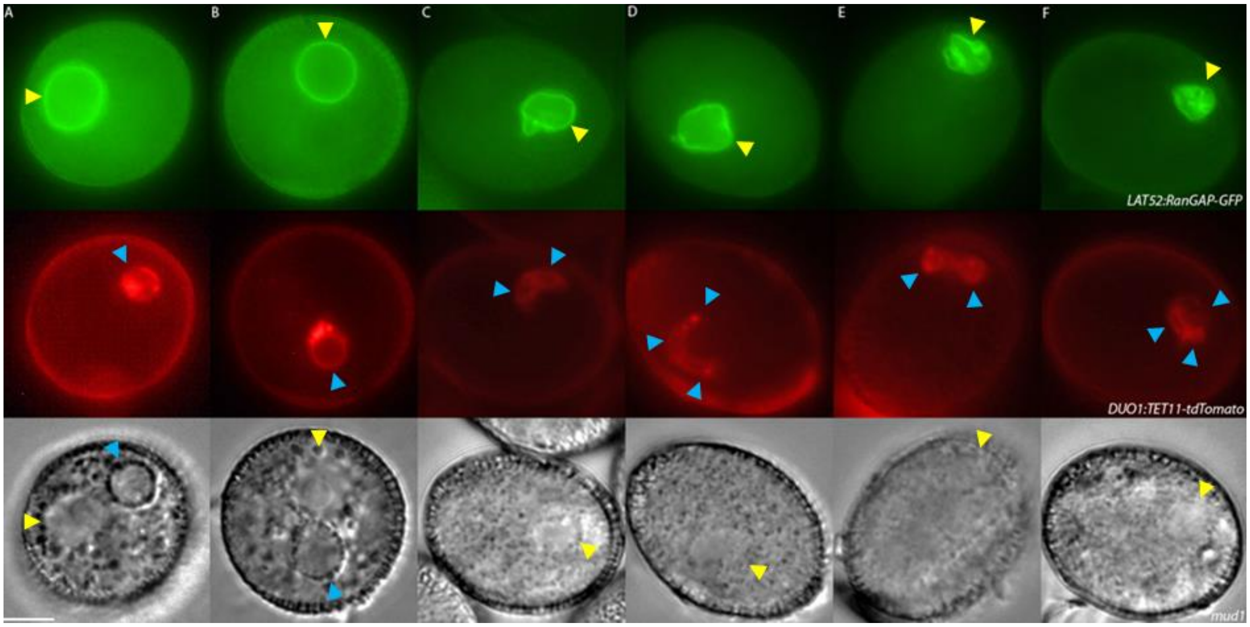

Figure 4. Light and fluorescence micrographs of *mud1*$^{-/-}$ *DUO1:TET11-tdTomato×LAT52:RanGAP-GFP* at different developmental stages. The upper row represents GFP (LAT52:RanGAP-GFP-tags VN nuclear membrane), middle RFP (DUO1:TET11-tdTomato-tags male germline plasma membrane) and lower DIC images. The VN (A-F), GC (A-B) or SCs (C-F) and MGU (A-F) are in the periphery of pollen at different bud stages. The intact MGU is shifted from the centre toward the periphery and positioned near the pollen wall in *mud1*(C-F). The VN appears to be displaced from the centre to the periphery of the pollen grain at the bicellular and tricellular bud stages (A-F). Displacement of the MGU from the centre to the periphery does not affect the association between the SC pair and VN and both structures are connected physically (MGU). Images are arranged from early to mature bud stages. Arrow heads show position of male germline (blue) or VC nuclear membrane (yellow) inside the pollen grain. Scale bar =5µm

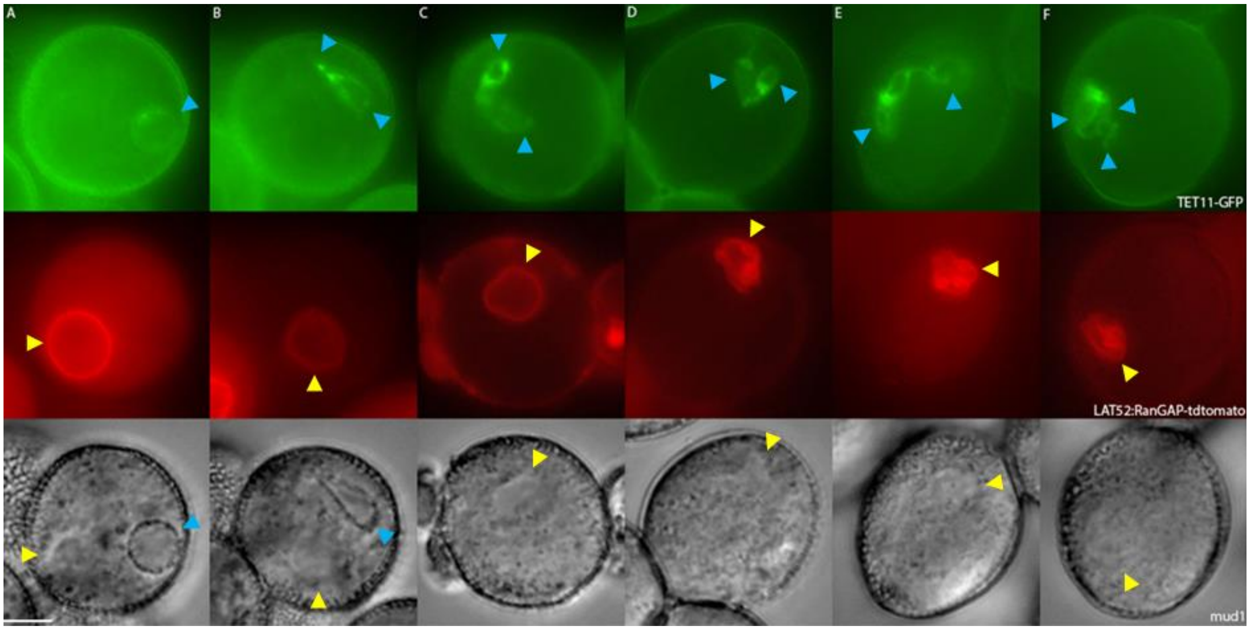

Figure 5. Light and fluorescence micrographs of *mud1*$^{-/-}$ *TET11-GFP×LAT52:RanGAP-tdTomato* at different developmental stages. The upper row represents GFP (TET11-GFP-tags male germline plasma membrane), middle RFP (LAT52:RanGAP-tdTomato-tags VN nuclear membrane) and lower DIC images. The VN (A-F), GC (A-B) or SCs (C-F) and MGU (A-F) are in the periphery of pollen at different bud stages. The MGU is displaced from the centre toward the periphery and positioned near the pollen wall (C-F). The VN appears to be displaced from the centre to the periphery of the pollen grain at the bicellular and tricellular bud stages (A-F). Displacement of the MGU from centre to periphery does not affect the association between the SC pair and VN and both structures relate to each other physically (MGU). Images are arranged from early to mature bud stages. Arrow heads show position of male germline (blue) or VC nuclear membrane (yellow) inside the pollen grain. Scale bar =5µm

Reviewer #2: comments on the MS_Plant Direct: 2024-01482TW and authors response

	**Reviewer-2 comments**	**Authors Response**
	**All updated changes are highlighted in Red in the MS suggested by the Reviewer please**	
**01**	Overall, the main weakness is the lack of quantitative data, as the consequence this work comes as highly descriptive.	Now we added the number of independent lines analyzed and pollen per stage, which can provide the quantitative data as suggested.
**02**	For example, when author says "Length of the cytoplasmic projection increases with GC body morphogenesis and an	You are right that there is no quantitative data on cytoplasmic projection, and our results are based on the observation of

	elongated cytoplasmic projection has been observed at the late bicellular stage (Fig. 1b, Fig. S2-S3)",	cytoplasmic projection initiation and its growth with pollen development, that the length increases. There were a few limitations to measuring the actual length of cytoplasmic projection-<br>1) Cytoplasmic projection has a folded shape.<br>2) Mostly it is not in one plane to measure its length.
**03**	Two cytoplasmic projections of equal length (20%) in A. thaliana." Is it possible to quantify this?	The data about cytoplasmic projections is published in our previous paper (Rauf et al., 2023).<br>Note: Serial 03_Additional information
**04**	It would be useful to show the different development stages and/or to include a scheme to facilitate the interpretation of them.	Dear Reviewer, each pollen represents an independent developmental stage shown in the figures. An explanation has been added to the legends of all the figures as you suggested, please. Furthermore, a schematic has been shown in Fig. 6 as suggested.
**05**	Finally, in my opinion the conclusions section does not reflect the main findings.	The conclusion section is updated as suggested.

Serial 03_Additional information

Genotype	Number of cytoplasmic projections	Developmental stage			
		GC		SC	
		Mean (SD)	%	Mean (SD)	%
WT (*N* = 3)	1	91.3 (26.3)	95.1	95.7 (48.0)	87.0
	2	4.7 (0.6)	4.9	14.0 (9.7)	13.0
duo1-1 (*N* = 3)	1	158.7 (119.4)	97.3	143.0 (109.4)	91.1
	2	4.3 (0.6)	2.7	14.0 (14.8)	8.9
duo1-3 (*N* = 3)	1	107.0 (11.8)	96.7	178.3 (80.6)	92.9
	2	3.7 (0.6)	3.3	13.7 (15.0)	7.1

Table S7. Frequency of germline cytoplasmic projections in wild type (WT) and *duo1* mutant pollen in GC and SC stage flower buds. Mean, standard deviation (SD) and percentage data are

shown for plants (N = 3) of each genotype, where 58 – 300 pollen were scored per plant for WT (HTR10:GFP-TUA6) or *duo1* (TET11-GFP). In WT pollen at SC stage, one SC always possessed a long cytoplasmic projection, while the second SC lacked a projection (87 %) or had a shorter projection (13 %). In chi-squared tests the proportion of pollen with one or two cytoplasmic projections was not significantly different between WT and *duo1* alleles at GC stage (P=0.51) or at SC stage (P=0.29). An increase in the proportion of pollen with two cytoplasmic projections between GC and SC stage was observed for all genotypes, but this was only significant (P<.05) for *duo1-1* (P=.012). (Rauf et al., 2023).
